# Physiological and genomic basis of mechanical-functional trade-off in plant vasculature

**DOI:** 10.3389/fpls.2014.00224

**Published:** 2014-05-28

**Authors:** Sonali Sengupta, Arun Lahiri Majumder

**Affiliations:** Division of Plant Biology, Acharya J C Bose Biotechnology Innovation Centre, Bose InstituteKolkata, India

**Keywords:** embolism, cavitation, xylem, drought, freezing, mechanical stress

## Abstract

Some areas in plant abiotic stress research are not frequently addressed by genomic and molecular tools. One such area is the cross reaction of gravitational force with upward capillary pull of water and the mechanical-functional trade-off in plant vasculature. Although frost, drought and flooding stress greatly impact these physiological processes and consequently plant performance, the genomic and molecular basis of such trade-off is only sporadically addressed and so is its adaptive value. Embolism resistance is an important multiple stress- opposition trait and do offer scopes for critical insight to unravel and modify the input of living cells in the process and their biotechnological intervention may be of great importance. Vascular plants employ different physiological strategies to cope with embolism and variation is observed across the kingdom. The genomic resources in this area have started to emerge and open up possibilities of synthesis, validation and utilization of the new knowledge-base. This review article assesses the research till date on this issue and discusses new possibilities for bridging physiology and genomics of a plant, and foresees its implementation in crop science.

## Introduction

A green plant is unique in its hydraulic architecture. Hydraulic conductivity of the xylem is closely linked to the minimum leaf area, which it must supply with water and nutrients for survival. Hydraulic conductivity, as quantified by Zimmermann (1974), is generally measured as leaf specific conductivity (flow rate per unit pressure gradient) divided by the leaf area supplied by the xylem pipeline segment. This measure is a key for quick evaluation of pressure gradients within a plant. Modeling the functional and natural architecture of plant water flow pipeline takes more traits in consideration than merely the physical attributes of a mechanical pump. The contribution of living cells and more specifically, genes and proteins, for maintenance of the “green pump” remains largely unaddressed.

Several theories have been proposed to explain ascent of sap. The operation of the green pump is simple yet elegant and is best described by the Cohesion-Tension Theory (CTT) (Dixon, 1914) but also synthesized from the work of many scientists over the last few decades. Besides physical explanations, the living parenchyma cells around xylem were originally proposed to be of importance by Bose (1923) in his pulsation theory. Later, the living xylem parenchyma cells indeed proved of high importance for the continuous ascent of sap.

**Figure 1:**
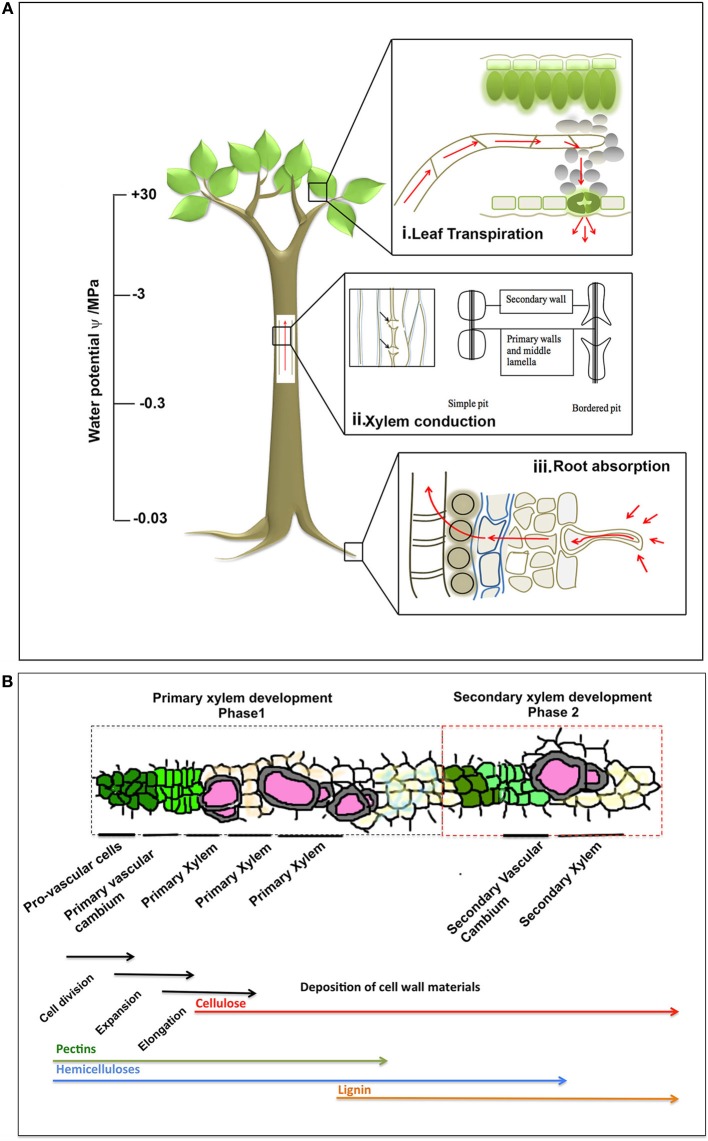
**(A)** The soil-plant-air continuum functioning in maintenance of water transport column. The plant root takes up water from soil, and the water column is maintained continuous along the xylem. The continuity across the xylem vessel is maintained by several intrinsic physical properties of water, input from the adjoining living cells and transpirational pool. The rough estimate of pressure along the vascular cylinder is presented in the scale bar (image not to actual scale). **(B)** A schematic of xylogenesis, adapted and modified from Hertzberg et al., 2001. The two phases of xylem development (primary and secondary); and the tissues involved in the process are shown within respective dotted boxes. The biological processes (cell division, expansion, elongation, deposition of cell wall) involved are shown by black arrows, under corresponding tissue types. The cell wall materials that are deposited are also shown under corresponding tissue types during xylogenesis. The order of such differentiation may be traced from left to right in the figure, though their actual time frame may differ from species to species.

The major governing factors are the physical properties of aqueous solution, means of transport and xylem anatomy, consideration of all of which makes the “sap conducting system” comparable to basic hydraulic systems such as pumps and irrigations in household or human blood vasculature. Components of such system are mainly (i) a driving force, (ii) a pipeline system, (iii) a reservoir and other regulating factors. To establish a soil-water-atmosphere continuum, an uninterrupted “water network” is necessary, which is built in the plant where transpirational evaporation is the driving force (Figure 1A). The evaporation of water from the porous green tissue surface creates a capillary pull in the water menisci (Figure 1Ai) and a curvature is induced in them, which is sufficient to support a huge water column against gravity in the stem and root vascular cylinder (Figure 1Aii). The water reservoir is the soil, wherefrom the root draws its supply (Figure 1Aiii). The empirical Jurin law says that a menisci radius of 0.12 μm can support a column of 120 m (Sperry, 2013; Zimmermann, 1983). The pull creates sub-atmospheric pressure in the xylem vessels. As the height of a plant increases, the water potential drops, and it is expected that leaves, twigs and upper extremities will display a 10–1000 times drop of pressure (Figure 1A, Tyree and Sperry, 1989). Sixty five percentage of the water potential drop occurs in tree trunk xylem, with a 20% contribution from root and 14% from leaves (Tyree and Sperry, 1989). This explains why big tree trunks can survive severe localized damages near the base.

## Plant architecture and the green pump

Architecture of a plant is defined by its height, girth, woodiness, root system design and shoot disposition. Such architecture varies across the plant kingdom, along which varies the plants' hydraulic nature. Secondary thickening is a major player that governs the green pump. It has been shown that root pressure plays little or no part in maintenance of this column in woody plants. Severing the root may not hamper upward movement of water, if there is a direct supply to the vessels; however leaves are necessary. Even the best vacuum pump is able to pull water to not more than 10.4 m, considering that a Sequoia tree may have to pull water up to 100 m. However, in the monocots, root pressure is considered to be a major player of sap pull.

Considering the physical properties of green-pump, cavitation and embolism are major threats to the water column in xylem and subsequently, to survival, across the kingdom. To successfully transport water and minerals from soil to leaf, existing pressure in xylem conduits needs to remain sub-atmospheric (negative), in contrast to animal system where long distance transport is actively under positive pressure. The molecular property of cohesion gives a high strength to water. Ultrapure water confined to tubes of very small bore will need a tension comparable to the strength needed to break steel columns of the same diameter. Cohesion imparts strength comparable to solid wires in a water column. The vice is: once air is introduced in such system, the column will snap apart. To prevent such snapping, xylem properties play an important role.

## Physiology of xylogenesis: the biphasic development in xylem

The biphasic development of xylem in plants is critical to understand the hydraulic architecture as well as the air-water-soil continuum (Figure 1B). Procambium develops into xylem precursor cells that eventually differentiate into xylem fiber cells, xylem parenchyma, and tracheary elements, consisting of vessels and tracheids in the first phase. The second phase deposits secondary xylem walls onto the primary xylem walls (Fukuda, 1997; De Boer and Volkov, 2003), derived from vascular cambium and made of cellulose microfibrils impregnated with lignin, structural proteins, hemicellulose and pectin (Figure 1B, Ye, 2002; Fukuda, 2004; Yokoyama and Nishitani, 2006). Prior to secondary development, the tracheary components elongate and with the advent of secondary wall deposition, the cellular components in the living tracheid undergo programmed cell death (Fukuda, 2004) living only the hollow pipeline (Fukuda, 1997; Zhang et al., 2011) composed of vessels interconnected by pits (De Boer and Volkov, 2003; Choat and Pittermann, 2009). The paired pits are often bordered (Figure 1A); from secondary deposition forming two overarched secondary walls, in between which a fine pit membrane with small pores persist. Pit membranes are made up of meshes of polysaccharide (Tyree and Zimmermann, 2002; Pérez-Donoso et al., 2010) and allow axial passage of water and small molecules. Besides, they act as safety protection against spread of air seeds (Tyree and Zimmermann, 2002; De Boer and Volkov, 2003; Choat et al., 2008; Pérez-Donoso et al., 2010).

## Physiology of cavitation

The negative pressure in the xylem may descend low enough to make the water metastable. To achieve non-disrupted flow in such system, water must remain liquid below its vapor pressure. This metastable state induces nucleation of vaporization, or cavitation. Cavitation is the introduction of air spaces into the continuous water column and under physical metastable state water is prone to form air bubbles easily. Introduced in a xylem lumen, air cavities rupture the water column and in its worst, block the transport of water and minerals to the leaf. This blockage is known as “embolism” and may lead the plant to a lethal fate.

Cavitation is known to occur in plants frequently. Paradoxically, occurrence of cavitation is the strongest support for CTT. It is only natural to observe cavitation if water is under such negative pressure. The root vessels of field grown, well watered maize plants have been known to embolize daily and then refill. Vessels that were filled by dawn may embolize at mid-afternoon and by sunset they are again refilled (McCully et al., 1998). When transpiration rate is high and water scarcity is at bay, trees display cavitation, which means that embolism can well be induced by water stress. Large metaxylem vessels show a higher rate of embolism, and evidence suggest that water stress-induced embolism is of the frequent most sort (Tyree and Sperry, 1989). It is a prerequisite for cavitation that some vessels are embolized to start with; which is met by bubbles introduced in some of the vessels by mechanical damage, harbivory and insect attack.

## Stress-induced embolism in plants

Both abiotic and biotic stresses can induce embolism in a plant. Drought and frost—induced embolisms are most prevalent, while mechanical stress and pathogen-induced damage are often the primary inducers.

Desert plants and dry-season crops are most threatened by drought-induced embolism. Air-seeding increases during drought as the sap pressure becomes increasingly negative due to high suction. The evaporation from leaf surface increases and the porous conduit wall may release air inside the functional conduits. They behave as nucleation centers and cause the sap pressure to increase to atmospheric level. The bubble is then likely to start an embolism that fills up the diameter of conduit, as the surrounding water is pulled up by transpiration.

Interconduit pit membranes with nano-scale pores normally restrict passage of air bubble from affected to functional conduits but at a high pressure difference they fail to stop the propagation. The rate of this propagation is important to measure the cavitation resistance in a plant.

Freezing is another cause of embolism, specially in woody temperate species. Freeze-thaw cycles may lead to 100% loss of water transport due to embolism in some species (Scholander et al., 1961). The primary governing factor in damage intensity seems to be the mean diameter of the conduits. Smaller vessel diameters are more vulnerable to damage.

Frost-induced air seeding is caused by segregation of gas by ice. There is a certain amount of salting out from the sap during freezing of sap, and if the salts are not able to move through the walls, they raise the osmotic pressure of remaining solution (Sevanto et al., 2012). This embolism can be more severe if there is functional drought prevailing. Freezing-induced embolism is a primary stress in forests where seasonal freeze-thaw is observed. Herbaceous plants, on the other hand, hardly survive freezing and are mostly at threat from drought-induced embolism.

Vascular wilt pathogens can wipe out entire crop. It is known that vascular pathogens induce water stress in their hosts; but can embolism be a cause of such stress? All vascular wilt pathogens break into rigid secondary xylem walls to enter the vessels as well as the pit membranes. Generally vascular wilt pathogens or their spores and conidia are too large to pass through pit membrane pores (Mollenhauer and Hopkins, 1974; Choat et al., 2003, 2004; Qin et al., 2008). Even when they manage to break into the vessel the milieu is not friendly. The microenvironment of xylem pipeline is nutritionally very poor and the pathogens surviving in xylem niche are not too many in number. It is speculated that they prefer this environment to minimize competition. Nevertheless, fungal and bacterial pathogens can extract the little amount of ions and nutrients available in the xylem stream and are able to break through and digest secondary wood to leech nutrition from living cells. Doing so, they weaken the pressurized cell wall and their infestation within the dead pipeline makes the water stream reactive and prone to cavitation. They may as well block the vessels and pit membranes, occluding parts of functional conduit network.

There is also an internal mechanical stress associated with ascent of sap. The high negative tension within the xylem pipeline causes an inward pool. Depending on the sapwood elasticity, there is a daily diameter change of tree trunk correlated to transpiration and daylight. In Scots pine, Perämäki et al. (2001) described daily changes in the sapwood diameter. The pull causes pressure on a stem surface element directed toward the center of the stem and the tracheal structure resists the movement of the surface element. The mechanical strength of the tracheary wall and its composition is, hence, an important factor in maintaining normal xylem activity as is the plasticity of pit membrane structure and composition.

## Vulnerability of xylem to cavitation

Xylem seems to be vulnerable to cavitation in many different ways. This vulnerability can vary depending on the species, season, and availability, state and temperature of water. Broadly, the vulnerability of plants to cavitation is often plotted on xylem vulnerability curves, which is a function of decline in xylem hydraulic conductivity due to increasingly negative xylem pressure. Such declines are typically expressed relative to the maximum decline possible as the Percentage Loss of Conductivity (PLC). Comparisons of the vulnerability to cavitation among species are made using the xylem pressure at 50% loss of conductivity (P_50_) with the traditional plotting of vulnerability curve (Meinzer and McCulloh, 2013). There remain controversies related to the techniques used for measurement of vulnerability described elsewhere in details (McElrone et al., 2012; Cochard et al., 2013; Wheeler et al., 2013).

The vulnerability curve for a number of tree species, as put forward by Tyree et al. (1999) shows a typical exponential shape, indicating that sub-zero pressure is a direct inducer of cavitation. This makes cavitation a regular process and necessitates a resistance mechanism in plants. It has also been claimed that cavitation is rapidly repaired by a miraculous mechanism (Holbrook and Zwieniecki, 1999) known as “refilling.” We can thus categorize cavitation resistance under two proposed mechanisms; one, by refilling the air bubbles efficiently; and two, by modulating pit membrane properties. The possible genetic controls of both are worthy of discussion.

## Cavitation resitance by refilling: a questionable trait

The removal of air seeds from lumen to turn a non-functional vessel to functional is known as refilling. The idea, though widely observed, recently was confronted with a serious doubt voiced by the plant hydraulic scientists. The long-established experimental procedure that has been followed to measure cavitation has been pronounced faulty (Sperry, 2013). It has been claimed that the standard procedure of xylem hydraulic conductivity measurement, by excising the stem under water to avoid air aspiration in the open conduits, is not a valid observation procedure. It has been suggested that in many species, significant amount of cavitation is introduced even when the stem is cut under water. The consequences of this artifact on previous datasets were significant, as it may be reflected in all vulnerability to cavitation curves obtained in other species for a long period of time; and perturb our analysis of refilled vessels.

However debatable the issue may be, recent high resolution and real-time imaging studies (Holbrook et al., 2001; Windt et al., 2006; Scheenen et al., 2007; Brodersen et al., 2010) also satisfy the requirements of the hypothesis that plant has some kind of resistance strategies to protect itself from embolism. It has been proposed that plants have an osmotically driven embolism repair mechanism and existing rehydration pathways through the xylem. The mechanisms were predicted to be largely of two types: (i) “novel” refilling, a refilling mechanism without “positive root pressures, even when xylem pressures are still substantially negative”; (ii) root pressure aiding the refilling of vessels raising the pressure inside vessels near atmospheric (Salleo et al., 1996; Holbrook and Zwieniecki, 1999; Tyree et al., 1999; Hacke and Sperry, 2003; Stiller et al., 2005). The first type is common among woody dicots whereas evidence of the second type is common among annual herbaceous species.

## Genetic control of refilling mechanism

Bay leaf tree, *Laurus nobilis* is an aromatic shrub in which mechanism of refilling is proposed to be linked to starch to sugar conversion. Reserve carbohydrate depletion from xylem parenchyma induces phloem unloading in a radial manner via ray parenchyma (Salleo et al., 2009; Nardini et al., 2011). Xylem-phloem solute exchange has been found to occur along both symplastic and apoplastic paths (Van Bel, 1990). It has been hypothesized that solutes might move radially along the ray cell walls, enter the embolized xylem conduits and increase the solute concentration of the residual water within them, thus promoting xylem refilling by altering osmoticum. The role of xylem parenchyma in refilling is significant. Lianas, shrubs and vine fibers are often observed to have living protoplasts and starch granules (Fahn and Leshem, 1963; Brodersen et al., 2010). Repeated cycles of embolism and repair are correlated to cyclic depletion of starch in xylem during drought (Salleo et al., 2009; Secchi et al., 2011). Debatably, repeated cycles of embolism formation and repair may disable the refilling mechanism and ultimately lead to carbon starvation (Sala et al., 2010, 2012; McDowell, 2011). The hydrolyzed starch movement from xylem is yet unresolved.

Water stressed *Populus trichocarpa* plants revealed an upregulation of ion transporters, aquaporins, and carbon metabolism related genes (Secchi et al., 2011; Secchi and Zwieniecki, 2012). A putative sucrose-cation co-transporter may aid the refilling process as suggested by the chemical profiling of vessel lumen. Grapevine refilling petioles show strong upregulation of carbon metabolism and aquaporin expression (Perrone et al., 2012).

A basic assumption is made that in dicots, to enhance refilling ability trait, one might target carbohydrate metabolizing genes in a localized manner to improve sucrose release. Sucrose may be used as an osmoticum inside non-functional lumens or may be used as energy currency. Localization of increased aquaporins (PIPs and TIPs) within axial parenchyma surrounding conduits may prove important. It is now proved by imaging studies (Brodersen et al., 2010) that living cells play a central role in embolism refilling and restoring transport, and by further prevention of air seed and pathogen by sealing off conduits with tyloses. Further detailed work is needed to identify the stress signals that mediate talk between xylem vessels and parenchyma.

In monocots, root pressure is the most important mechanism for refilling reported till date. Grasses exhibit root pressure more often, and with the increase of plant height the basal root pressure increases (Cao et al., 2012). Monocots do not exhibit secondary thickening and ray cells thus the osmoticum and sucrose transport theory do not apply to monocots (Andre, 1998). Selection for root pressure in these species solves the embolism repair problem and negates the need for carbohydrate transport along the pathway common in woody angiosperms (Brodersen et al., 2013). However, Stiller et al. (2005) showed the presence of “novel” refilling in rice in presence of high negative pressure and suggested that in upland or low-rainfed rice this mechanism can serve side by side of a positive root pressure. Root pressure may involve a stronger mechanical tissue, and whether or not any trade-off between safety and efficiency is involved is unclear. Study of more vascular function mutants in monocot crops may resolve the genes involved in this process.

## Genomic perspective: genes, proteins and models implicated in refilling

**Figure 2:**
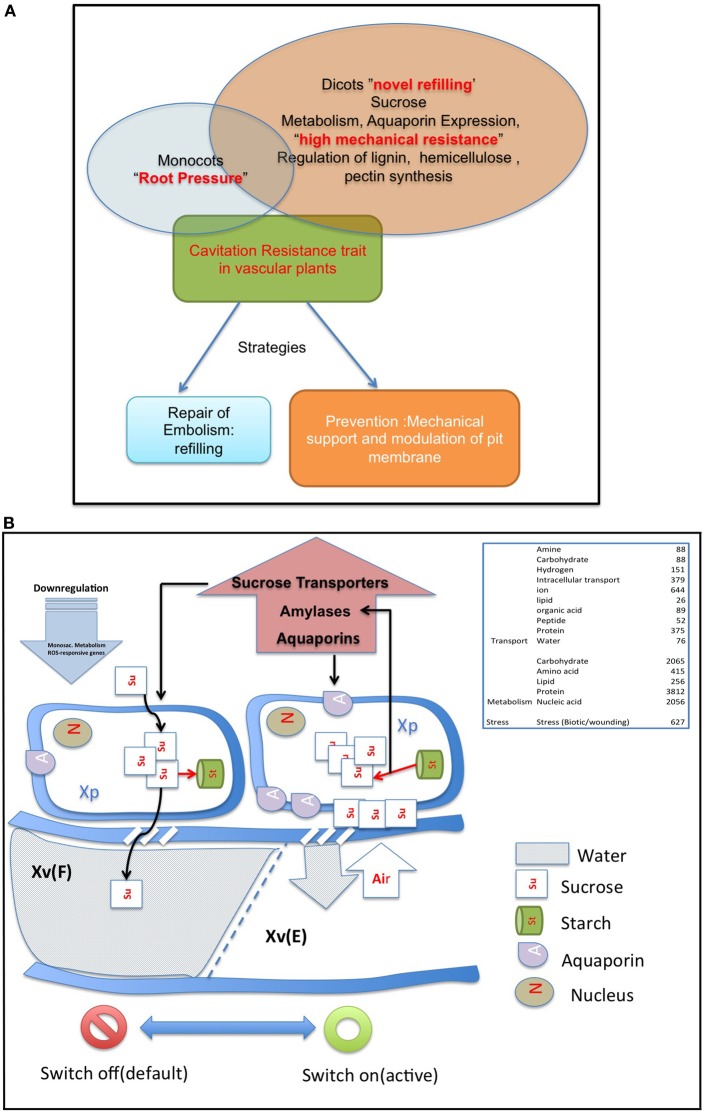
**(A)** The strategies of vascular plant in a battle against embolism. Monocots often employ root pressure, while dicots employ novel refilling mechanism, and mechanical resistance to resist cavitation. There is no clear demarcation between the strategies employed by the two groups, and the strategies may overlap. **(B)** The sugar sensing model of embolism refilling process, modified from Secchi et al. (2011). For detail explanations of the model, refer text and Secchi et al. (2011). Briefly, when vessels are filled and functional, a default “switch off” mode is active. Sucrose is continuously transported from accompanying xylem parenchyma cells into the vessels. Cavitation induces a “switch on” mode of sensing. When a vessel is filled with air, free passage of sucrose to the vessel lumen is hindered, and the sucrose molecules are deposited on vessel wall. This, with a positive feedback loop generates a cascade of high starch to sucrose conversion (Bucci et al., 2003; Salleo et al., 2004; Regier et al., 2009). The increased sucrose pool would be maintained by upregulation of amylases and sugar transporters. The genes up/downregulated during the sensing process are mentioned in the figure. Abbreviations used: Xv(F), Xylem Vessel Filled; Xv(E), Xylem Vessel Embolized; Xp, Xylem Parenchyma. Other abbreviations are explained in the figure.

The battle with cavitation is fought either with efficient refilling or fine structural modulation of pit membrane and strength of vascular cylinder wall. The genomic, transcriptomic and proteomic studies may thus come under two broad sections: genomic basis of refilling and genomic basic of mechanical strength (Figure 2A).

## Genomic basis of refilling

The process of refilling or repair of embolism requires pumping water in an air-filled cavity. Physically this will require an empty or air-filled vessel, functional neighbor vessels, a source of energy to drive the refilling and a source of water to refill. In the previous sections, the physical and physiological components of embolism repair have been discussed in detail. However, a reductionist biologist looks further beyond for the possible identities of molecular candidates that repair the non-functional vessel. It is hypothesized that refilling is a result of an intricate interaction of xylem parenchyma, (even possibly phloem), vessel wall chemistry, and the composition and flexibility of pit membranes (Holbrook and Zwieniecki, 1999). The signals that are sensed when embolism occurs and the cascades that follow the primary signal transduction event, involve interconnected molecular regulators; that has been subject of several studies. The most recent model of refilling puts forward a role of sugar signaling in embolism sensing and refilling mechanism, the involved gene families being Aquaporins, Sucrose transporters and enzymes related to starch breakdown, Alpha and Beta Amylase (Secchi and Zwieniecki, 2010).

## Aquaporins

**Figure 3:**
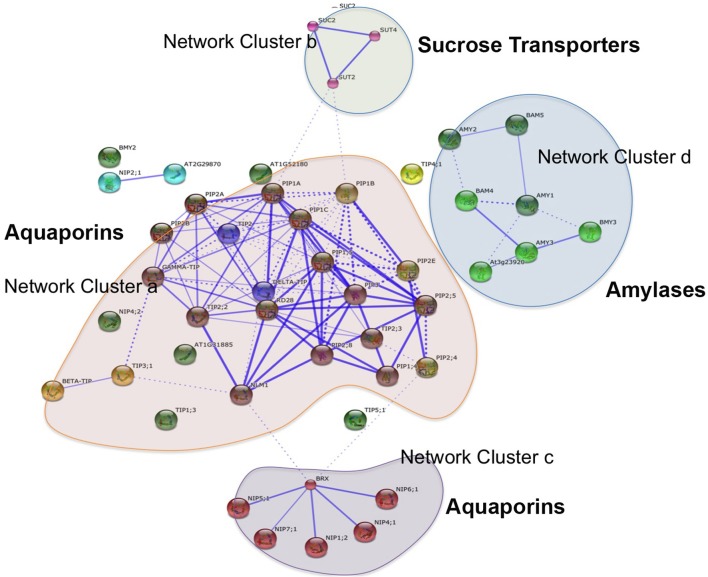
**The protein-protein interaction network of Arabidopsis sucrose transporters, amylases and aquaporins, generated using String database**. Thicker lines indicate stronger reaction (Szklarczyk et al., 2010).

**Figure 4:**
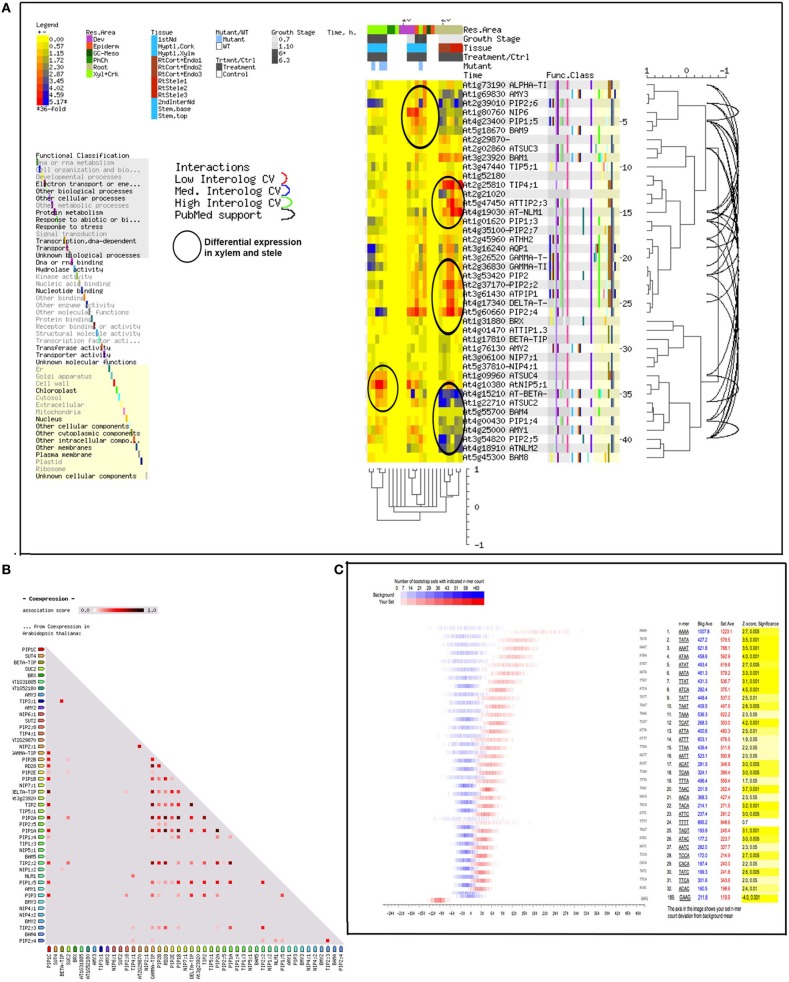
**(A)** Localization of the genes from Tables 1, 2 in various Arabidopsis tissue, from public microarray databases, and e-northern tool at Botany Array Resource (Toufighi et al., 2005). **(B)** Co-expression profile of the genes in Arabidopsis (Szklarczyk et al., 2010). **(C)** Distribution of relevant n-mers in the promoters of the above genes. That may induce shared expression. The results are generated using String and Promomer tools in Botany Array Resource (Toufighi et al., 2005). A tabulated form of the results are presented in Supplementary Table 5.

Aquaporins are conservedly implicated in the refilling process of angiosperms and gymnosperms from the very beginning. The refilling of vessels in *Populus trichocarpa* is accompanied by selective upregulation of PIPs (Plasma Membrane Intrinsic Proteins). Secchi et al. (2011) proposed that the sensing of embolism and accomplishment of refilling is mediated by sugar signals, specifically sucrose. According to their proposed model, when a vessel is filled with air, free passage of sucrose to the vessel lumen is hindered, and the sucrose molecules are deposited on vessel wall. This, with a positive feedback loop generate a cascade of high starch to sucrose conversion (Bucci et al., 2003; Salleo et al., 2004; Regier et al., 2009). The increased sucrose pool would be maintained by upregulation of amylases and sugar transporters. Secchi et al. (2011) showed a distinct upregulation in aquaporins and sucrose transporter (*PtSuc 2.1*) in air injected or artificially high osmotica-treated vessels. *Ptsuc2.1* shows a high homology to walnut sucrose transporter, which, on upregulation is able to relieve freeze-thaw induced embolism (Decourteix et al., 2006). The increased sucrose and the upregulation of aquaporins are correlated spatially and temporally, but connections are difficult to establish. The model hence proposed is schematically represented in Figure 2B. Almeida-Rodriguez et al. (2011) showed a gene expression profile of 33 Aquaporins in fine roots of hybrid poplar saplings and compared light and high transpiration induced vascular hydraulics physiology with respect to Aquaporin expression. Dynamic changes were observed in expression pattern of at least 11 aquaporins from poplar; and some of them were localized in the root tissue. In Arabidopsis, Postaire et al. (2010) showed that, hydraulic conductivity of excised rosettes and roots are correlated wih expression of aquaporins. AtPIP1; 2, AtPIP2;1, and AtPIP2;6 are the most highly expressed PIP genes in the Arabidopsis rosette (Alexandersson et al., 2005) and under long night, AtPIP1;2 knockout plants loose 21% hydraulic conductivity in the rosette(Postaire et al., 2010). The disturbed hydraulics phenotype is a genetic dissection of the direct relation between aquaporin expression and plant water transport; although there may be components other than Aquaporin that may serve an important role (Sack and Holbrook, 2006; Heinen et al., 2009). It has been shown in hybrid poplar *Populus trichocarpa* × *deltoides*, increasing evaporation from leaf surface and perturbed hydraulics is correlated with high aquaporin expression (Plavcová et al., 2013). In common grapevine, *Vitis vinifera L*. (cv Chardonnay) inhibitors of aquaporin-mediated transport greatly affects both leaf hydraulic conductance and stomatal conductance (Pou et al., 2013). Of 23–28 Aquaporin isoforms in grapevine, a subset including VvPIP2;2, VvTIP1;1 plays important role during early water stress, while VvPIP2;1, VvPIP2;3, VvTIP2;1 are highly expressed during recovery(Pou et al., 2013). In Maize roots, radial water transport are diurnally regulated by proteins from the PIP2 group (Lopez et al., 2003). It is evident, though, that not all aquaporins participate in the refilling process. The sugar signal initiation is one important component; as originally described by Secchi et al. (2011) and must induce embolism-related aquaporin isoforms. The transcriptomic studies show that a very high number of Carbohydrate Metabolism related genes were upregulated during embolism (Secchi et al., 2011). Upregulation of the disaccharide metabolism gene group was observed, along with downregulation of monosaccharide metabolism; that suggests an accumulation of sucrose pool on the vessel wall (Secchi et al., 2011). Further upregulation of ion transporters and downregulation of carbohydrate transporters build up an osmoticum inside the cell to facilitate efflux of water. Figure 2B (inset) shows a summary of the number of gene categories showing differential expression during embolism (Secchi et al., 2011). The energy required for the pumping in comes from starch hydrolysis and one can presume, xylem specific isoforms of aquaporin, Starch synthetase and sucrose transporters will be highly expressed during refilling in plants. For critical evaluation of the model parameters, and its feasibility across the plant kingdom we extracted all aquaporin gene sequences from Arabidopsis and the Arabidopsis homologs of *Populus trichocarpa* sucrose transporters and amylases implicated in embolism Secchi et al., 2009, 2011; Secchi and Zwieniecki, 2010, 2012, 2013, 2014. The accession numbers of the fetched Arabidopsis genes are presented in Tables 1A,B. We subjected the gene sequences to protein-protein interaction network interaction analysis in String software in Expasy, without suggested functional neighbors (Szklarczyk et al., 2010). Generated interaction network for Arabidopsis gene subsets (mentioned in Table 1) clearly shows three interaction network clusters, connected to each other (Figure 3), the middle cluster (termed ‘a’ in Figure 3) shows evidenced network of PIPs as well as a RD28, dehydration stress related protein. Two other clusters (b and c in Figure 3) exhibit sucrose transporters and NIPs. Amylases form an un-joined node (d in Figure 3). We further localized the genes in Arabidopsis publicly available transcriptome analysis database in different tissues and observed shared enrichment in root endodermis, cortex and stele using e-northern (Figure 4A, Toufighi et al., 2005). A co-expression profile (Figure 4B) was obtained using string software, and the common n-mers present in the genes to induce a co-expression in certain tissues has been analyzed using promomer tool (Figure 4C; Table 2, Supplementary Table 1, Toufighi et al., 2005). Many of the enriched *cis*-elements contribute to dehydration and sugar stress. Overall, the genomic and transcriptomic data and candidate-gene based data emphasizes the high probability of sugar sensing of embolism. Secchi and Zwieniecki (2014) also showed that in hybrid poplar, downregulation of PIP1 delimits the recovery of the plant from water-stress-induced embolism, and thus is probably manages the vulnerability of xylem in negative pressure under control condition. The sugar content in the plant tissue strengthens the view further (Secchi and Zwieniecki, 2014).

**Table 1A:** **Genes, families and members important in refilling experimentally reported in *Populus trichocarpa***.

**Gene families**	**Specific genes**				
	**Family**	**Subfamily**	**Gene name**	**JGIv2.0 annotation name**	**Arabidopsis homologs**
Aquaporins	PIP (Plasma Intrinsic Protein)	PoptrPIP1	PoptrPIP1.1	POPTR_0008s06580	For analysis, the entire aquaporin family of Arabidopsis has been used instead of only specific homologs, refer to Table 1B.
			PoptrPIP1.2	POPTR_0003s12870	
			PoptrPIP1.3	POPTR_0010s19930	
			PoptrPIP1.4	POPTR_0006s09920	
			PoptrPIP1.5	POPTR_0016s12070	
		PoptrPIP2	PoptrPIP2.1	POPTR_0006s09910	
			PoptrPIP2.2	POPTR_0009s13890	
			PoptrPIP2.3	POPTR_0004s18240	
			PoptrPIP2.4	POPTR_0016s09090	
			PoptrPIP2.5	POPTR_0010s22950	
			PoptrPIP2.6	POPTR_0006s12980	
			PoptrPIP2.7	POPTR_0008s03950	
			PoptrPIP2.8	POPTR_0009s01940	
Alpha-beta amylases	Alpha-amylase	PoptrAMY	PtAMY1	POPTR_0515s00220	AT4G25000
			PtAMY2	POPTR_0002s01570	AT1G76130
			PtAMY3	POPTR_0010s10300	AT1G69830
	Beta amylase	PoptrBMY	PtBMY1a	POPTR_0008s17420	AT3G23920
			PtBMY1b	POPTR_0001s11000	AT3G23920
			PtBMY2	POPTR_0003s10570	AT5G45300
			PtBMY3	POPTR_0008s20870	AT5G18670
			PtBMY4	POPTR_0003s08360	AT2G02860
			PtBMY5	POPTR_0017s06840	AT1G09960
Sucrose transporters	Sucrose transporter		PtSUC2.1	POPTR_0019s11560	AT5G55700
			PtSUT1.2	POPTR_0013s11950	AT4G15210
			PtSUT2.a	POPTR_0008s14750	AT1G22710

*Gene ID data compiled from Secchi et al. (2011); TAIR and phytozome public database*.

**Table 1B:** **The entire aquaporin family in Arabidopsis extracted from TAIR**.

**Gene family name**	**Accession**	**TIGR Protein Type**
Delta tonoplast integral protein family	At1g31880	Major intrinsic protein, putative
	At1g80760	Nodulin-like protein
	At1g73190	Tonoplast intrinsic protein, alpha (alpha-TIP)
	At2g45960	Aquaporin (plasma membrane intrinsic protein 1B)
	AT3g06100	Putative major intrinsic protein
	AT5g47450	Membrane channel protein-like; aquaporin (tonoplast intrinsic protein)-like
	AT3g53420	Plasma membrane intrinsic protein 2a
	At2g36830	Putative aquaporin (tonoplast intrinsic protein gamma)
	At2g37170	Aquaporin (plasma membrane intrinsic protein 2B)
	At2g37180	Aquaporin (plasma membrane intrinsic protein 2C)
	AT4g35100	Plasma membrane intrinsic protein (SIMIP)
	At2g29870	Putative aquaporin (plasma membrane intrinsic protein)
	At1g01620	Plasma membrane intrinsic protein 1c, putative
	AT3g61430	Plasma membrane intrinsic protein 1a
	AT3g54820	Aquaporin/MIP–like protein
	At1g17810	Tonoplast intrinsic protein, putative
	AT3g47440	Aquaporin-like protein
	At2g16850	Putative aquaporin (plasma membrane intrinsic protein)
	At2g39010	Putative aquaporin (water channel protein)
	AT3g16240	Delta tonoplast integral protein (delta-TIP)
	At1g52180	Aquaporin, putative
	AT4g23400	Water channel–like protein
	At2g25810	Putative aquaporin (tonoplast intrinsic protein)
	AT4g00430	Probable plasma membrane intrinsic protein 1c
	AT5g37810	Membrane integral protein (MIP)–like
	AT5g37820	Membrane integral protein (MIP)–like
	AT4g17340	Membrane channel like protein
	AT4g10380	Major intrinsic protein (MIP)–like

**Table 2:** **Representative common n-mer details over represented in the embolism with respective transcription factors and their probable roles**.

**n-mers**	**Z-score**	**Regulation mode**	**Probable role**	**Consensus matches to n-mer in the PLACE 25.0.1 database**
AAAT^**^	3.5	Positive	Dehydration responsive	Matched AAAT at offset 4 in CACTAAATTGTCAC 14BPATERD1: “14 bp region” (from −599 to −566) necessary for expression of erd1 (early responsive to dehydration) in dehydrated Arabidopsis
ATAA^**^	4.0	Positive	Sugar responsive	Matched ATAA at offset 2 in ACATAAAATAAAAAAAGGCA −314MOTIFZMSBE1: located between −314 and −295 region of maize (Z.m.) Sbe1 gene promoter; critical positive cis element; important for the high-level, sugar-responsive expression of the Sbe1 gene in maize endosperm cells; recognized by nuclear protein
ATAT^**^	2.7	Positive/negative	MADS domain	Matched [AT][AT][AT][AT] at offset 5 in TTDCCWWWWWWGGHAA AGAMOUSATCONSENSUS: binding consensus sequence of Arabidopsis (A.t.) AGAMOUS MADS domain
AATA	3.3	Positive	Sugar-responsive	Matched AATA at offset 6 in ACATAAAATAAAAAAAGGCA −314MOTIFZMSBE1: Located between −314 and −295 region of maize (Z.m.) Sbe1 gene promoter; critical positive cis element; important for the high-level, sugar-responsive expression of the Sbe1 gene in maize endosperm cells; recognized by nuclear protein
TTAT	3.1	Positive	Sugar responsive, binding activity to Myb core	Matched AATA at offset 6 in ACATAAAATAAAAAAAGGCA −314MOTIFZMSBE1: located between −314 and −295 region of maize (Z.m.) Sbe1 gene promoter; critical positive cis element; important for the high-level, sugar-responsive expression of the Sbe1 gene in maize endosperm cells; recognized by nuclear protein; matched TATT at offset 2 in TTTATTTACCAAACGGTAACATC23BPUASNSCYCB1: “23 bp UAS (Upstream activating sequence)” found in the promoter of *Nicotiana sylvestris* (N.s.) CycB1 gene; located between −386 and −409; contains a 5 bp element identical to the MYB binding core (ACGT); required for M-phase-specific expression; binds protein complexes in a cell cycle-regulated manner
ATCA^**^	4.5	Positive/negative	MADS domain, homeobox binding domain	Matched [AT][AT][ACGT][ACGT] at offset 8 in NTTDCCWWWWNNGGWAAN AGL1ATCONSENSUS: binding consensus sequence of Arabidopsis (A.t.) AGL1 (AGAMOUS-like 1); AGL1 contains MADS domain; see S000339; AGL20 is a MADS domain gene from Arabidopsis that is activated in shoot apical meristem during the transition to flowering; AGL20 is also regulated by the Gibberellin pathway; complex regulatory net works involving several MADS-genes underlie development of vegetative structures
GAAG^**^	4.0	Positive	ABA-responsive, MADS	Matched GAAG at offset 6 in ATGTACGAAGC ABAREG2: motif related to ABA regulation; gene: sunflower helianthinin; transacting factor: bZIP? Matched [ACGT][ACGT][AT][ACGT] at offset 0 in NNWNCCAWWWWTRGWWAN AGL2ATCONSENSUS: binding consensus sequence of Arabidopsis (A.t.) AGL2 (AGAMOUS-like 2); AGL2 contains MADS domain; AGL2 binds DNA as a dimer
CGAA	2.4	Positive	ABA-responsive	Matched CGAA at offset 5 in ATGTACGAAGC ABAREG2: motif related to ABA regulation; gene: sunflower helianthinin; transacting factor: bZIP?

*An html table for all n-mers is presented in Supplementary Table 1*.

** *denotes overrepresentation*.

## Transcription factors

The corregulation of sugar metabolism and water transport pathways require a complex transcriptional switch. Indeed, a large number of transcription factors control the refilling process, and they may regulate the diurnal pattern, the temporal accuracy and spatial distribution of the pathways involved. The role of TFs is shared; However, a look at the *cis* elements of pathway components may elucidate the nature of such sharing. The transcription factors important for xylogenesis and probably embolism are: AP2/EREBP, bZIP, C3HHD-ZIPIII, NAC, MYB, bHLH, WRKY, AP2/ERF, WRKY, HD, AUX/IAA, ARF, ZF, AP2, MYC, (Arabidopsis); HD-ZIPIII, MYB, MADS, and LIM in *Populus*, MYB and Hap5a in Pine and HRT in *Hordeum* (Dharmawardhana et al., 2010). With the onset of genomic approaches, much more intensive analysis have been made possible. In a comprehensive genome-wide transcriptome analysis of *P. trichocarpa*, with snapshots from each elongating internode from a sapling stage (Internode1 through Internode11) a large number of differential representation of transcription factors have been obtained (Dharmawardhana et al., 2010). No less than 1800 transcription factors were readily detectable in at least one growth phase, of which, 439 are differentially regulated during xylogenesis (Dharmawardhana et al., 2010); some of which are represented in Table 3. Another study identified 588 differentially changed transcripts during shoot organogenesis in *Populus* (Bao et al., 2009, 2013). While the refilling process is majorly governed by sugar and dehydration signaling, NAC and Myb TF families remain singularly important in both xylem maturation and lignin biosynthesis. Aspects of xylogenesis that may be linked with mechanical-functional trade-off of vascular bundle revolve around lignin. There have been studies on genomics and transcriptomics of xylogenesis and secondary wood formation; however the genes responsible to maintain integrity of the vascular cylinder are not clearly known. In Supplementary Table 2, a comparative snapshot of some selected transcripts and emanating studies revealing the xylogenesis transcriptome in gymnosperms and angiosperms is provided. Several recent studies address the genomics of xylogenesis excellently; some of which are summarized in Table 4.

**Table 3:** **Some representative transcription factors in *Populus trichocarapa* Xylem Maturation (Dharmawardhana et al., 2010)**.

WRKY family transcription factor
DRE binding protein (DREB1A)
Ethylene responsive element binding factor
Putative AP2 domain transcription factor
Ethylene responsive element binding factor 4 (aterf4,9)
Homeodomain–like protein.1
Auxin response transcription factor (ARF1,9)
WRKY family transcription factor
ATPAO4 (POLYAMINE OXIDASE 4); amine oxidase
Ethylene-responsive transcriptional coactivator
Lateral root primordia (LRP1)
Transcription factor TINY, putative
MADS-box protein
Putative CCCH-type zinc finger protein
bHLH protein/contains helix-loop-helix DNA binding motif
Zinc finger protein Zat12
WRKY family transcription factor
BEL1-like homeobox 4 protein (BLH4)
TINY-like protein
Myb family transcription factor
Putative squamosa-promoter binding protein
Putative transcription factor/similar to transcription factor SF3
ES43 like protein/ES43 protein
AP2 domain protein RAP2.1
Abscisic acid responsive elements-binding factor (ABF3)
bHLH protein/contains helix-loop-helix DNA binding motif
Myb family transcription factor
CCAAT-binding transcription factor subunit A (CBF-A)

**Table 4:** **Representative transcriptome studies in literature**.

**Xylogenesis**	**Embolism**	**Lignin biosynthesis**
Li et al., 2013	Secchi et al., 2011	Hertzberg et al., 2001
Carvalho et al., 2013		Zhong et al., 2011
Pesquet et al., 2005		Lu et al., 2005
Li et al., 2012		Schrader et al., 2004
Dharmawardhana et al., 2010		
Karpinska et al., 2004		
Bao et al., 2009		
Rengel et al., 2009		
Mishima et al., 2014		
Plavcová et al., 2013		
Zhong et al., 2011		

## Cavitation resistance introduced by pit membrane

The major key of cavitation resistance is pit membrane adaptation. To survive, ultrastructure of pit membrane needs to balance between minimizing vascular resistance and limiting invasion by pathogen and microbes. While the first is favored by thin and highly porous membrane, the later needs thick membrane and narrower pores. This calls for a trade-off between water transport function and biotic invasion resistance.

The thickness range of the pit membranes in the angiosperms is very broad, almost 70–1900 nm and so are the diameter of the pores (10–225 nm). Species with thicker pit membrane and smaller pores prevent seeding and embolism more successfully and thus may represent the group of species which has higher drought resistance.

Pit membrane porosity is not the only determinant of air bubble propagation among conduits. The other factor which serve equally important role is the contact angle between pit membrane and air water interface. This particular property is a direct function of pit membrane composition. The more hydrophobic the membranes are the more the contact angle and subsequently lower the pressure needed for air-seeding. Additionally, high lignin content, though required for mechanical strength, interrupt with the hydrogeling of pectins. Pectic substances can swell or shrink in presence or absence of water and thus they control the porosity of membranes. Polygalacturonase mutants in *Arabidopsis* showed a higher P_50_ value (−2.25MPa), suggesting a role for pectins in vulnerability to cavitation (Tixier et al., 2013). Mechanically stronger pit membranes thus may resist stretching and expansion of pore membranes indicating a compromise in function. Water stress has been reported to exhibit a direct relation to low lignin synthesis (Donaldson, 2002; Alvarez et al., 2008) although it is not known whether this low lignin help the water transport better.

## Suggested genetic basis of cavitation resistance by pit membrane modulation and mechanical support

Identification of genes and proteins behind the structural and mechanical controls of pit membrane formation has not progressed so far as repair mechanism of embolism is concerned. Genetic aspects of plant hydraulics are little studied, since most of the xylem studies are done in woody trees and study of herbaceous crops is rather scant. It is hard to obtain mutants in trees as the generation time is high, and the study process is long and laborious. Also, hydraulics in plants is not a simple structural or functional trait but is a complex physiological phenomenon. Figuring out the multitrait control switch of this function is thus difficult.

## Can lignin biosynthesis be considered as a control switch?

**Figure 5:**
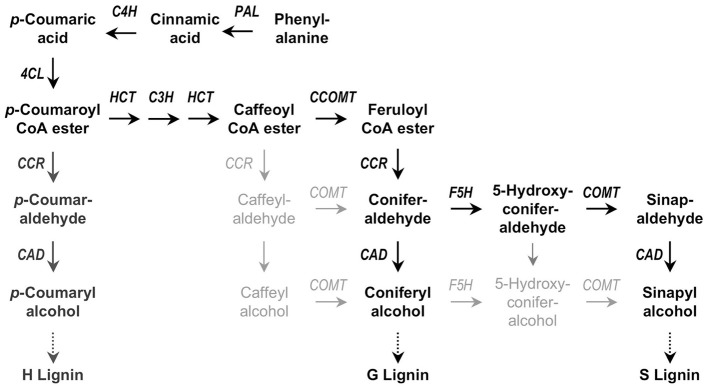
**Simplified scheme for monolignol synthesis**. The main pathway in dicotyledonous plants is highlighted in black, involving phenylalanine ammonia-lyase (*PAL*), cinnamate 4-hydroxylase (*C4H*), 4-coumarate CoA ligase (*4CL*), *p*-hydroxycinnamoyl-CoA: quinate shikimate *p*-hydroxycinnamoyltransferase (HCT), *p*-coumarate 3-hydroxylase (*C3H*), caffeoyl-CoA *O*-methyltransferase (*CCOMT*), hydroxycinnamyl-CoA reductase (*CCR*), ferulate 5-hydroxylase (*F5H*), caffeate *O*-methyltransferase (COMT), and cinnamyl alcohol dehydrogenase (*CAD*). Alternate pathways are in light gray. H subunits are only minor lignin components in dicots. Adapted from Quentin et al. (2009).

Among the living cell processes that may take active part in controlling hydraulics, lignin biosynthesis is a major candidate and highly deciphered. In chemical nature, it is a polymer of phenylpropanoid compounds synthesized through a complex biosynthetic route (Figure 5; Hertzberg et al., 2001; Vanholme et al., 2010). Luckily enough, the genes on the metabolic grid are sequenced in plants like *Arabidopsis* and *Populus*, which is helpful to understand their modulation under stress. Till date, both biotic and abiotic stressors have been implicated in modulation of lignin biosynthesis, as well as seasonal, developmental and varietal changes (Anterola and Lewis, 2002; Zhong and Ye, 2009). Representing a large share of non-fossil organic carbon in biosphere, lignification provides mechanical support and defends the plant against pests and pathogens. The mechanical support, further, is mostly linked to xylem vessels and hydraulics.

Lignin is made from monolignols (hydroxy-cinnamyl alcohol), sinapyl alcohol, coniferyl alchol, and *p*-coumaryl alcohol in a smaller quantity. The complex metabolic grid and the transcriptional switches are described in details elsewhere (Hertzberg et al., 2001). The major metabolic pathway channeling into this grid is phenylpropanoid pathways through phenylalanine (Phe). Phe, synthesized in plastid through shikimic acid biosynthesis pathway, eventually generates p-coumaric acid by the activity Phenylalanine Ammonia-Lyase (PAL) and Cinnamate 4-Hydroxylase (C4H). *p*-coumaric acid empties itself into the lignin biosynthesis grid to result into three kinds of lignin units; guaiacyl (G), syringyl (S), and p-hydroxyphenyl (H) units. Gymnosperm lignin polymer is majorly composed of G and H units, angiosperms show G and S units and H is elevated in compressed softwood and grasses (Boerjan et al., 2003).

There are stresses in nature that change plant lignin content. For example, lignin amount in *Picea abies* is predicted to correlate positively with annual average temperature (Gindl et al., 2000). Temperate monocots as well show an increase of lignin in response to increasing temperature (Ford et al., 1979). In *Triticum aestivum*, 2°C chilling stress decreases leaf lignin but increases in root is observed (Olenichenko and Zagoskina, 2005). Curiously, some studies have shown that although no changes in the levels of lignin or its precursors were observed in plants maintained at low temperatures, there was an increase in related enzyme activities as well as an increase in gene expression. Cold acclimatization in Rhododendron shows upregulation of C3H, a cytochrome P450-dependent monooxygenase without further functional characterization (El Kayal et al., 2006). It has been argued that expression of *C3H* could result in changes in the composition of lignin, altering the stiffness of the cell wall albeit without a definitive proof. The basal part of the maize roots show a growth reduction and low plasticity of cell wall associated with upregulation of two genes in lignin grid (Fan et al., 2006) in response to drought. The increase of free lignin precursors in the xylem sap and reduced anionic peroxidase activity in maize has been associated with low lignin synthesis in drought (Alvarez et al., 2008). It is possible that reducing lignin may directly affect the vascular tissue, encouraging water transport, lowering air seeding and increasing cavitation resistance; however it is not known what share of reduced lignin actually amount to stem vasculature, water column support and pit membrane plasticity.

## Biotechnological modification of lignin metabolism

With the advancement of genomic data, it is now possible to map the genetic changes which may influence hydraulic architecture. However, the model systems are questionable. Among the woody plant species, the genome of poplar has been sequenced; and the lignin biosynthesis network is fully characterized in *Arabidopsis* and rice. It is expected that change in lignin content may result differently in herbaceous and woody plants. There are controversial results obtained so far. In free-standing transgenic poplar trees, a 20–40% reduction in lignin content was associated with increased xylem vulnerability to embolism, shoot dieback and mortality (Voelker et al., 2011). Similarly the severe inhibition of cell wall lignification produced trees with a collapsed xylem phenotype, resulting in compromised vascular integrity, and displayed reduced hydraulic conductivity and a greater susceptibility to wall failure and cavitation (Coleman et al., 2008). A study on the xylem traits of 316 angiosperm trees in Yunnan, and their correlations with climatic factors claimed that wood density and stem hydraulic traits are independent variables (Zhang et al., 2013).

A weak pipeline and less lignification compromises vascular integrity as observed from the above results. On the other hand, low lignin helps to increase the plasticity of the pit membrane pectin. Thus compromising lignin quantity may have serious impact on strength of the vascular cylinder; on the other hand, it may increase the pit membrane hydrophilic property and may offer resistance toward cavitation.

Lately, *Arabidopsis* has been taken in as a model for secondary tissue development, although it lacks formation of secondary wood. Tixier et al. (2013) argued that *Arabidopsis* might be as well considered to be a model of xylem hydraulics. They regarded the inflorescence stem of *A. thaliana* as a model for xylem hydraulics despite its herbaceous habit, as it has been shown previously that the inflorescence stem achieves secondary growth (Altamura et al., 2001; Ko et al., 2004), allows long-distance water transport from the roots to the aerial parts of plant, and experience gravity and other mechanical perturbations (Telewski, 2006). There are distinct similarities between woody dicots and *Arabidopsis* inflorescence stems with respect to vessel length and diameter as well as presence of simple perforation plates and border (Sperry et al., 2005; Hacke et al., 2006; Schweingruber, 2006; Wheeler et al., 2007; Christman and Sperry, 2010). It has a genetic potential to develop ray cells and rayless wood is observed in juvenile trees (Carlquist, 2009; Dulin and Kirchoff, 2010). Having *Arabidopsis* as a full proof model for woodiness may open numerous possibilities. The best among them are study of environmental stresses on hydraulic characters. A number of mutants can be generated and screened in *Arabidopsis* with deviant safety vs. efficiency phenotype with little effort. The *Arabidopsis thaliana* irregular xylem 4 phenotype (*irx4*) a mutant for cinnamoyl-CoA reductase 1 (*CCR1*) gene, has provided us with valuable insight in the role of lignin reduction and associated phenotypic changes in vasculature. As reported by Jones (2001), near-half decrease of lignin component with no associated change in cellulose or hemicellulose content gives the plant an aberrant vascular phenotype. Most of the cell interior is filled up with expanded cell wall and the xylem vessels collapse. Abnormal lignin gives the cell wall a weak ultrastructure and less structural integrity (Jones et al., 2001; Patten et al., 2005). Later it has been claimed that by modulating the *CCR* gene, *irx4* mutant has obtained a delayed albeit normal pattern of lignification program (Laskar et al., 2006). It thus has to be borne in mind that not only the content but the spatio-temporal pattern of lignin deposition may change the xylem ultrastructure and change the safety-efficiency trade-off limit.

There are a few transcriptional control switches in lignin production which can be used in modification of vascular conductance. Modulation of co-ordinate expression of cellulose and lignin in rice is an important study regarding such transgene opportunities. Expression of the *Arabidopsis SHN2* gene (Aharoni et al., 2004) under a constitutive promoter in rice alters its lignocellulosic properties along with introduction of drought resistance and enhanced water use efficiency (Karaba, 2007). The *Arabidopsis* SHINE/WAX INDUCER (SHN/WIN) transcription factor belongs to the AP2/ERF TF family, and besides wax regulation, control drought tolerance in *Arabidopsis* (Aharoni et al., 2004; Broun et al., 2004; Kannangara et al., 2007). Expression analysis of cell wall biosynthetic genes and their putative transcriptional regulators shows that moderated lignocellulose coordinated regulation of the cellulose and lignin pathways which decreases lignin but compensates mechanical strength by increasing cellulose. All the processes ascribed to master control switch SHN may be directed toward evolution of land plants; waxy cover to lignin synthesis for erect disposition and water transport. However, no xylem irregularities are seen in this mutant (Aharoni et al., 2004).

As the best studied pathway related to secondary cell wall formation, lignin biosynthesis should offer the best metabolic grid that can be tweaked in plants to genetically understand mechanical functional trade-off and resistance to cavitation. General reduction of PAL (Phenylalanine ammonia lyase, E.C. 4.3.1.5) activities in developing plants may be one possible point of interest. PAL is a “metabolic branch- point” where Phe is directed toward either lignins or proteins (Rubery and Fosket, 1969). However, according to Anterola et al. (1999, 2002) and other such studies there are other pathways originating from pentose phosphate or glycolysis that may directly end into lignin biosynthesis and PAL may not serve as rate limiting step at all. Cinnamate 4-hydroxylase (C4H) is another candidate that has been downregulated with decrease in overall lignin content, however, with no effect on vascular integrity or function (Fahrendorf and Dixon, 1993; Nedelkina et al., 1999). *p*-Coumarate-3-hydroxylase (C3H) in *Arabidopsis* (CYP98A3) may be necessary and rate-limiting step in the monolignol pathway (Schoch et al., 2001). Its expression is correlated with the onset of lignification and a mutant line results in dwarfed phenotype with reduced lignin (Schoch et al., 2001). Cinnamoyl CoA O-methyltransferase (CCOMT), 4-coumarate:CoA ligase (4CL), cinnamoyl CoA reductase (CCR), and cinnamyl alcohol dehydrogenase (CAD) isoforms are downstream pathways in monolignol formation, and their relation to vascular integrity are yet to establish, though phenotypes associated with their mutations are tall/dwarf stature, altered lignin composition, and reduced mechanical support. Conclusive data are yet to be obtained from these studies.

## Conclusion

Hydraulic safety margin in a plant is clearly driven by its phylogenetic origin. Conifers have developed minimal hydraulic resistance which is a necessity for water transport through short unicellular tracheids. The unique torus-margo anatomy of the conifer pit membrane let them adaptively overpower multicellular vessels in angiosperms in certain cases. Conifer stems are proposed to have larger hydraulic safety margins when compared with most angiosperm stems (Meinzer et al., 2009; Choat et al., 2012; Johnson et al., 2012) although it is also suggested that they recover poorly from drought-induced embolism (Brodribb et al., 2010). The refilling mechanisms vary greatly between monocots and dicots and herbaceous and woody plants. Resistance to cavitation is thus closely related to many factors: such as nature of the mechanical tissue, the vasculature, the height of the plant, the systematic position of the plant, developmental stage and stresses the plant must face. It can be further emphasized that though, in certain dicots a trade-off within the water transport ability and mechanical strength (efficiency vs. safety) has been observed, the genomic factors which may control the trade-off are not identified till date completely; and the observation is far from universal. The two major physiological phenomena which seem to be linked to embolism resistance are lignification and solute transport between xylem parenchyma, vessel and phloem. The genes and proteins behind these physiological traits are many, and even the obtained transgenic plants and mutants have only been scantily characterized. The effects of assembly of the components are poorly understood and the models proposed do not address all plant families universally. Overall, although a phylogenetic trend is observed among the plants for the evolutionary establishment of hydraulic safety margins, the mechanisms behind have not been understood enough till date to predict the molecular basis and evolution in genomic scale. However, the best metabolic pathway to offer advantageous biotechnological outputs appears to be the lignin synthesis network, which should be assessed by mutant screening as well as by tissue specific overexpression studies in the plant. In case of monocots, drought-induced root- specific overexpression may be of advantage in generating better crops, as root pressure seems to be the major regulator. Crop biotechnology is largely benefitted when the gene pool and their interaction behind a biological process is better known. Overexpressing aquaporins along with the sugar sensing network under a dehydration-responsive promoter could be a formidable strategy to prevent embolism-induced wilting. An approach toward modulation of lignin biosynthesis grid regulation may yield better woody, or even herbaceous crops. The overwhelming knowledge emanating from transcriptomic and genomic studies build the platform where biologists can attempt crop modification for such complex traits as vascular integrity and water transport, without or marginally limiting other beneficial traits, in near future.

### Conflict of interest statement

The authors declare that the research was conducted in the absence of any commercial or financial relationships that could be construed as a potential conflict of interest.
