# Supplementary material for: Physiological and genomic basis of mechanical-functional trade-off in plant vasculature
Source: Front Plant Sci. 2014 May 28;5:224. doi: 10.3389/fpls.2014.00224 (PMC4035604; doi:10.3389/fpls.2014.00224)
Supplement: Supplementary file 1 [file DataSheet1.PDF]

BAR

Promomer n-mer Landscapes: Output for Promomer2

Opening local file /var/www/html/ntools/temp_general/promomer_1397588498_res.txt and drawing distributions...Copy the image by right-clicking it and then either copying it or saving it to your local computer. The graphic below indicates the background distribution in blue and your set distribution or value in red for the given n-mer. The colour-scale represents the Number of bootstrap sets with indicated n-mer count. The number of occurrences in your set are expressed relative to the mean of the background distribution, with the scale for this visible at the bottom of the page. Significant differences, based on a Z-score, are highlighted in yellow.. Click on the n-mer link beside the image to view the actual distribution graph for that n-mer.

Number of bootstrap sets with indicated n-mer count
0 7 14 21 29 36 43 51 58 >65
Background
Your Set

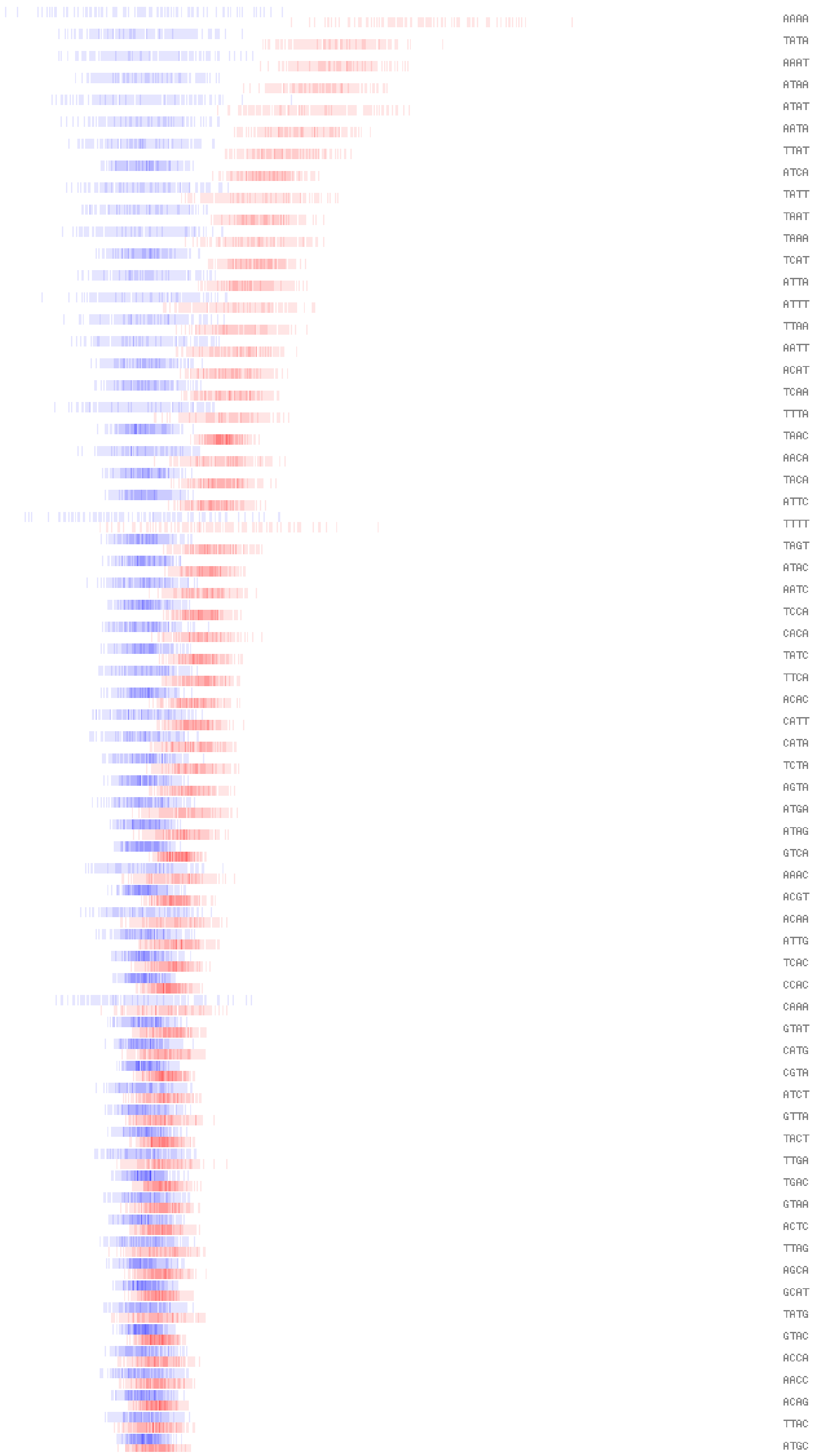

	n-mer	Bkg Ave	Set Ave	Z-score, Significance
1.	AAAA	1007.8	1223.1	2.7, 0.005
2.	TATA	427.2	579.5	3.5, 0.001
3.	AAAT	621.8	768.1	3.5, 0.001
4.	ATAA	458.6	592.9	4.0, 0.001
5.	ATAT	493.4	619.8	2.7, 0.005
6.	AATA	461.3	579.2	3.3, 0.001
7.	TTAT	431.3	536.7	3.1, 0.001
8.	ATCA	282.4	375.1	4.5, 0.001
9.	TATT	448.4	537.0	2.5, 0.01
10.	TAAT	409.5	497.5	2.8, 0.005
11.	TAAA	536.3	622.2	2.3, 0.05
12.	TCAT	268.3	353.0	4.2, 0.001
13.	ATTA	400.6	480.3	2.5, 0.01
14.	ATTT	603.1	676.5	1.9, 0.05
15.	TTAA	439.4	511.6	2.2, 0.05
16.	AATT	523.1	590.8	2.0, 0.05
17.	ACAT	281.5	346.9	3.0, 0.005
18.	TCAA	324.1	389.4	3.0, 0.005
19.	TTTA	496.4	559.4	1.7, 0.05
20.	TAAC	201.9	262.4	3.7, 0.001
21.	AACA	368.3	427.4	2.3, 0.05
22.	TACA	214.1	271.5	3.2, 0.001
23.	ATTC	237.4	291.2	3.0, 0.005
24.	TTTT	895.2	948.6	0.7
25.	TAGT	193.9	245.4	3.1, 0.001
26.	ATAC	177.2	223.7	3.0, 0.005
27.	AATC	282.0	327.7	2.3, 0.05
28.	TCCA	172.0	214.9	2.7, 0.005
29.	CACA	197.4	240.0	2.2, 0.05
30.	TATC	199.3	241.8	2.6, 0.005
31.	TTCA	301.6	343.6	2.0, 0.05
32.	ACAC	160.5	198.6	2.4, 0.01
33.	CATT	304.5	341.4	1.5
34.	CATA	256.3	293.0	1.6
35.	TCTA	210.4	246.9	2.0, 0.05
36.	AGTA	189.8	224.6	2.2, 0.05
37.	ATGA	266.8	298.4	1.6
38.	ATAG	180.8	209.5	1.9, 0.05
39.	GTCA	137.1	162.2	2.0, 0.05
40.	AAAC	416.6	440.0	0.8
41.	ACGT	111.9	135.2	1.7, 0.05
42.	ACAA	384.9	407.7	0.8
43.	ATTG	258.4	281.1	1.2
44.	TCAC	154.1	174.9	1.4
45.	CCAC	108.1	125.7	1.3
46.	CAAA	523.5	540.7	0.4
47.	GTAT	182.4	199.1	1.1
48.	CATG	168.2	184.0	1.0
49.	CGTA	87.4	102.8	1.3
50.	ATCT	257.6	272.1	0.8
51.	GTTA	206.9	220.9	0.8
52.	TACT	180.1	193.9	0.9
53.	TTGA	318.0	331.8	0.6
54.	TGAC	132.4	145.6	1.0
55.	GTAA	215.6	228.5	0.8
56.	ACTC	149.2	161.9	0.8
57.	TTAG	214.7	227.1	0.7
58.	AGCA	138.5	150.4	0.8
59.	GCAT	120.2	130.7	0.9
60.	TATG	215.7	225.7	0.6
61.	GTAC	86.2	96.0	0.9
62.	ACCA	191.2	200.9	0.6
63.	AACC	193.9	203.6	0.5
64.	ACAG	123.2	132.7	0.7
65.	TTAC	197.9	205.7	0.5

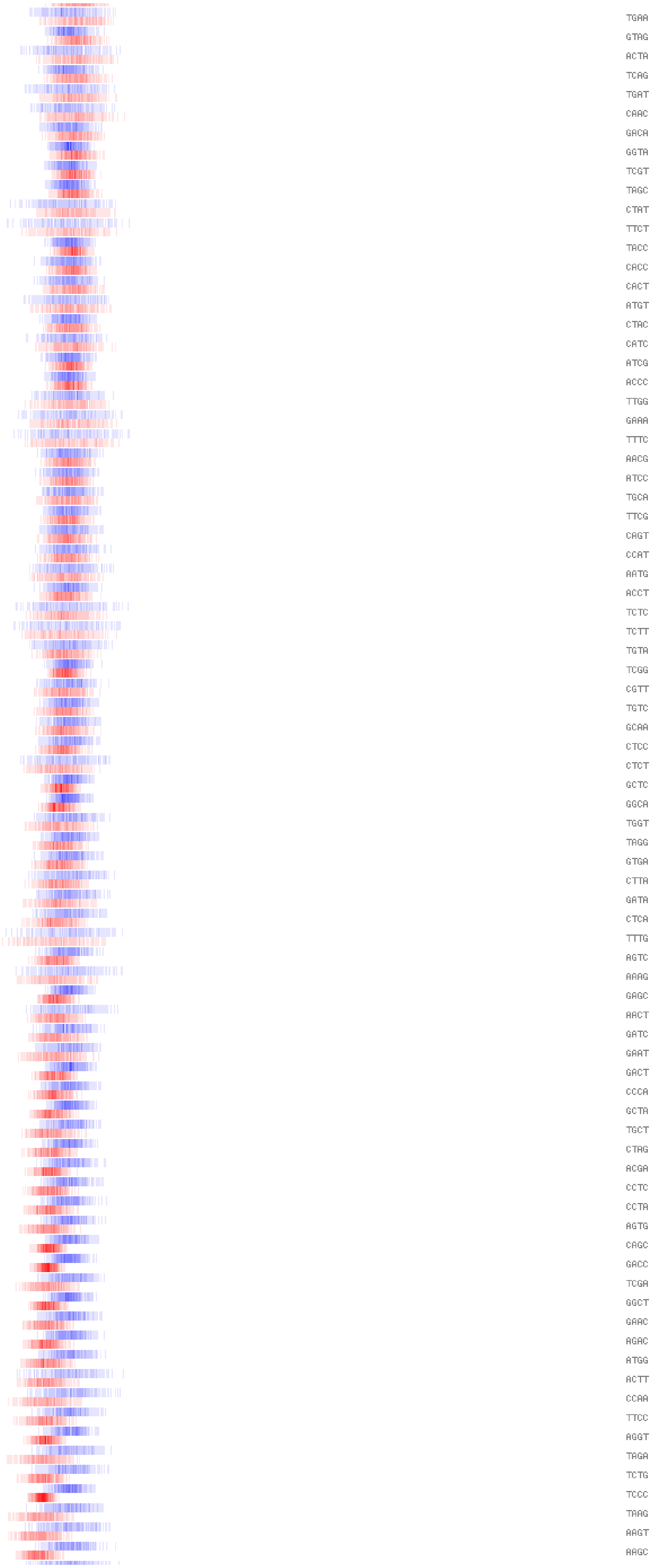

66.	ATGC	111.7	118.9	0.6
67.	TGAA	300.9	308.0	0.4
68.	GTAG	103.5	109.7	0.5
69.	ACTA	231.2	237.0	0.2
70.	TCAG	124.9	130.3	0.4
71.	TGAT	273.4	278.7	0.2
72.	CAAC	203.7	208.6	0.2
73.	GACA	143.2	147.9	0.3
74.	GGTA	94.7	98.7	0.4
75.	TCGT	113.6	117.5	0.3
76.	TAGC	95.6	99.0	0.3
77.	CTAT	224.0	227.3	0.1
78.	TTCT	358.4	361.4	0.1
79.	TACC	96.3	99.0	0.3
80.	CACC	101.0	103.3	0.2
81.	CACT	149.2	151.2	0.1
82.	ATGT	249.8	251.3	0.1
83.	CTAC	113.0	114.1	0.1
84.	CATC	180.4	181.2	0.0
85.	ATCG	104.3	104.9	0.1
86.	ACCC	83.1	83.5	0.0
87.	TTGG	205.7	205.9	0.0
88.	GAAA	403.2	403.4	0.0
89.	TTTC	371.3	370.6	-0.0
90.	AACG	127.0	126.2	-0.1
91.	ATCC	132.8	131.7	-0.1
92.	TGCA	134.0	132.8	-0.1
93.	TTCG	119.8	118.2	-0.1
94.	CAGT	117.9	115.8	-0.2
95.	CCAT	170.6	168.0	-0.2
96.	AATG	262.0	259.1	-0.2
97.	ACCT	124.9	121.8	-0.2
98.	TCTC	232.3	228.8	-0.1
99.	TCTT	370.9	366.7	-0.1
100.	TGTA	210.4	206.1	-0.2
101.	TCGG	68.6	64.2	-0.4
102.	CGTT	129.6	125.0	-0.3
103.	TGTC	136.2	131.5	-0.4
104.	GCAA	156.2	151.5	-0.3
105.	CTCC	109.1	102.8	-0.4
106.	CTCT	231.0	224.2	-0.3
107.	GCTC	79.3	72.4	-0.6
108.	GGCA	70.8	62.4	-0.8
109.	TGGT	173.3	164.8	-0.5
110.	TAGG	104.5	95.9	-0.7
111.	GTGA	148.6	139.9	-0.6
112.	CTTA	229.7	220.6	-0.5
113.	GATA	203.6	194.2	-0.6
114.	CTCA	177.8	166.9	-0.6
115.	TTTG	418.5	407.3	-0.4
116.	AGTC	132.0	120.7	-0.8
117.	AAAG	369.8	358.2	-0.5
118.	GAGC	77.5	65.8	-1.1
119.	AACT	288.7	276.6	-0.6
120.	GATC	147.4	135.1	-0.8
121.	GAAT	247.9	234.9	-0.8
122.	GACT	118.6	105.6	-1.1
123.	CCCA	111.5	97.6	-1.0
124.	GCTA	98.2	82.6	-1.4
125.	TGCT	126.4	110.6	-1.1
126.	CTAG	112.1	96.2	-1.3
127.	ACGA	129.9	113.0	-1.2
128.	CCTC	102.1	84.8	-1.3
129.	CCTA	114.8	97.5	-1.3
130.	AGTG	131.8	113.7	-1.3
131.	CAGC	78.7	60.5	-1.5
132.	GACC	78.0	59.1	-1.8, 0.05
133.	TCGA	129.6	110.6	-1.3
134.	GGCT	83.3	63.9	-1.7, 0.05
135.	GAAC	144.0	123.9	-1.4
136.	AGAC	136.3	116.1	-1.5
137.	ATGG	144.3	124.0	-1.4
138.	ACTT	280.2	259.4	-0.9
139.	CCAA	239.6	218.6	-1.0
140.	TTCC	163.6	142.2	-1.3
141.	AGGT	112.5	91.0	-1.8, 0.05
142.	TAGA	224.6	202.2	-1.2
143.	TCTG	141.6	118.2	-1.5
144.	TCCC	82.8	59.3	-2.1, 0.05
145.	TAAG	203.4	179.4	-1.4
146.	AAGT	270.1	244.2	-1.3
147.	AAGC	160.4	133.2	-1.8, 0.05

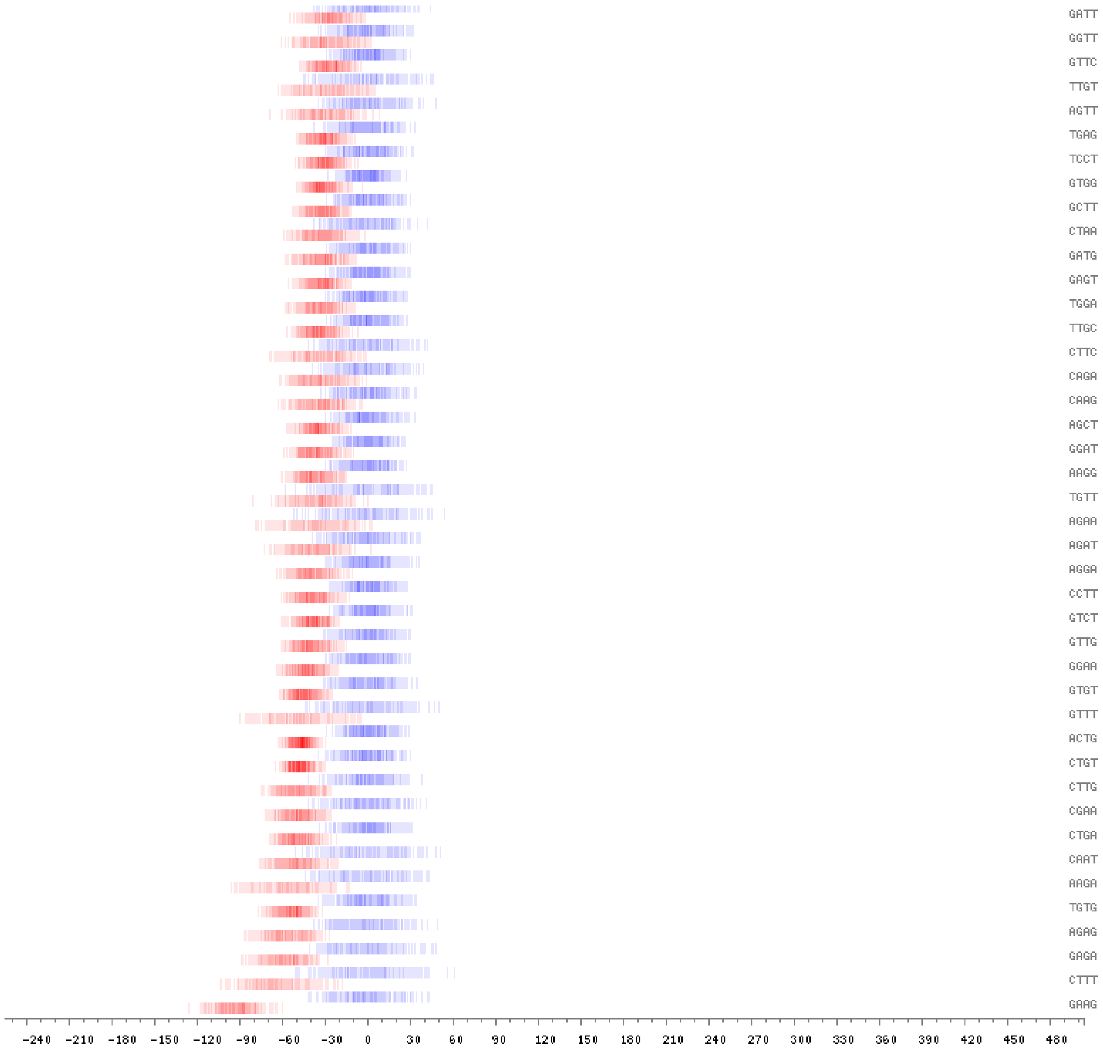

148.	GATT	286.2	258.9	-1.4
149.	GGTT	170.0	142.5	-1.6
150.	GTTC	145.6	117.6	-1.9, 0.05
151.	TTGT	339.9	311.6	-1.1
152.	AGTT	279.2	248.9	-1.5
153.	TGAG	163.9	133.4	-1.8, 0.05
154.	TCCT	145.7	114.4	-2.0, 0.05
155.	GTGG	106.3	74.7	-2.4, 0.01
156.	GCTT	154.3	121.9	-2.1, 0.05
157.	CTAA	260.1	227.5	-1.5
158.	GATG	155.5	122.7	-2.1, 0.05
159.	GAGT	136.4	103.5	-2.2, 0.05
160.	TGGA	175.1	142.1	-2.2, 0.05
161.	TTGC	146.0	112.2	-2.4, 0.01
162.	CTTC	218.9	185.0	-1.5
163.	CAGA	175.7	141.6	-1.7, 0.05
164.	CAAG	193.7	159.5	-1.9, 0.05
165.	AGCT	148.5	113.7	-2.3, 0.05
166.	GGAT	148.0	112.2	-2.5, 0.01
167.	AAGG	143.9	107.8	-2.4, 0.01
168.	TGTT	333.9	297.5	-1.3
169.	AGAA	384.4	347.6	-1.4
170.	AGAT	267.2	228.2	-2.0, 0.05
171.	AGGA	147.7	108.6	-2.5, 0.01
172.	CCTT	158.5	119.2	-2.6, 0.005
173.	GTCT	143.6	103.9	-2.7, 0.005
174.	GTTG	176.5	136.0	-2.4, 0.01
175.	GGAA	164.3	120.9	-2.8, 0.005
176.	GTGT	146.0	101.7	-2.7, 0.005
177.	GTTT	384.8	338.3	-1.8, 0.05
178.	ACTG	116.3	68.9	-3.6, 0.001
179.	CTGT	130.9	83.3	-3.0, 0.005
180.	CTTG	184.8	136.3	-2.8, 0.005
181.	CGAA	154.4	105.7	-2.4, 0.01
182.	CTGA	143.7	94.4	-3.1, 0.001
183.	CAAT	309.1	258.4	-1.8, 0.05
184.	AAGA	386.8	331.9	-2.0, 0.05
185.	TGTG	167.7	112.6	-3.1, 0.001
186.	AGAG	208.9	152.5	-2.4, 0.01
187.	GAGA	232.0	173.8	-2.3, 0.05
188.	CTTT	388.3	325.4	-2.2, 0.05
189.	GAAG	211.8	119.9	-4.0, 0.001

The axis in the image shows your set n-mer count deviation from background mean
