# Supplementary material for: Physiological and genomic basis of mechanical-functional trade-off in plant vasculature
Source: Front Plant Sci. 2014 May 28;5:224. doi: 10.3389/fpls.2014.00224 (PMC4035604; doi:10.3389/fpls.2014.00224)
Supplement: Supplementary file 2 [file DataSheet2.DOCX]

**Supplentary Table 2 . Representative genes important in xylogenesis of *P. trichocarpa* (representative angiosperm) and Zinnia sp.(representative gymnosperm)**

| **Metabolic and other genes for Xylem Maturation in Populus trichocarpa( Dharmawardhana et. al. 2010 )** |
| --- |
| **CCR1 (CINNAMOYL COA REDUCTASE 1)** |
| **CIP7 (COP1-INTERACTING PROTEIN 7)** |
| **MTHFR2 (METHYLENETETRAHYDROFOLATE REDUCTASE 2); methylenetetrahydrofolate reductase (NADPH)** |
| **ARAC2 (RHO-RELATED PROTEIN FROM PLANTS 7); GTP binding** |
| **ANAC073 (Arabidopsis NAC domain containing protein 73); transcription factor** |
| **DNA-binding family protein / remorin family protein** |
| **TUA4 (tubulin alpha-4 chain)** |
| **ATPAO4 (POLYAMINE OXIDASE 4); amine oxidase** |
| **ICK4/KRP6 (KIP-RELATED PROTEIN 6); cyclin binding / cyclin-dependent protein kinase inhibitor** |
| **unknown protein** |
| **PAL1 (PHE AMMONIA LYASE 1); phenylalanine ammonia-lyase** |
| **protease inhibitor/seed storage/lipid transfer protein (LTP) family protein** |
| **integral membrane family protein** |
| **unknown protein** |
| **PAL1 (PHE AMMONIA LYASE 1); phenylalanine ammonia-lyase** |
| **unknown protein** |
| **ATC4H/C4H/CYP73A5 (CINNAMATE 4-HYDROXYLASE, CINNAMATE-4-HYDROXYLASE); trans-cinnamate 4-monooxygenase** |
| **unknown protein** |
| **rhomboid family protein** |
| **AGP23/ATAGP23 (ARABINOGALACTAN-PROTEIN 23)** |
| **SHM4 (SERINE HYDROXYMETHYLTRANSFERASE 4); glycine hydroxymethyltransferase** |
| **caffeoyl-CoA 3-O-methyltransferase, putative** |
| **isoflavone reductase, putative** |
| **hydrolase, alpha/beta fold family protein** |
| **ATOMT1 (O-METHYLTRANSFERASE 1)** |
| **GAUT12/IRX8/LGT6 (GALACTURONOSYLTRANSFERASE 12); polygalacturonate 4-alpha-galacturonosyltransferase/ transferase, transferring glycosyl groups / transferase, transferring hexosyl groups** |
| **MAT2/SAM-2 (S-adenosylmethionine synthetase 2); methionine adenosyltransferase** |
| **remorin family protein** |
| **ALPHA-SNAP2 (ALPHA-SOLUBLE NSF ATTACHMENT PROTEIN); soluble NSF attachment protein** |
| **glycine-rich protein** |
| **COBL4/IRX6 (COBRA-LIKE4)** |
| **glycoside hydrolase family 28 protein / polygalacturonase (pectinase) family protein** |
| **IQD10 (IQ-domain 10); calmodulin binding** |
| **BTB/POZ domain-containing protein** |
| **glutaredoxin family protein** |
| **IAA10 (indoleacetic acid-induced protein 10); transcription factor** |
| **UTP--glucose-1-phosphate uridylyltransferase family protein** |
| **scarecrow-like transcription factor 8 (SCL8)** |
| **CAD4 (CINNAMYL ALCOHOL DEHYDROGENASE 4); cinnamyl-alcohol dehydrogenase** |
| **FLA12 (fasciclin-like arabinogalactan-protein 12)** |
| **ATMAP70-5 (microtubule-associated proteins 70-5); microtubule binding** |
| **ACL5 (ACAULIS 5)** |
| **ATGPX2 (GLUTATHIONE PEROXIDASE 2); glutathione peroxidase** |
| **glycosyltransferase family 14 protein / core-2/I-branching enzyme family protein** |
| **chitinase** |
| **RAP2.2; DNA binding / transcription factor** |
| **zinc finger (C3HC4-type RING finger) family protein** |
| **FLA12 (fasciclin-like arabinogalactan-protein 12)** |
| **FLA12 (fasciclin-like arabinogalactan-protein 12)** |
| **MTO3 (S-adenosylmethionine synthase 3); methionine adenosyltransferase** |
| **ATCIMS (COBALAMIN-INDEPENDENT METHIONINE SYNTHASE); 5-methyltetrahydropteroyltriglutamate-homocysteine S-methyltransferase/ methionine synthase** |
| **serine-rich protein-related** |
| **CESA4 (CELLULOSE SYNTHASE 4); transferase, transferring glycosyl groups** |
| **UDP-glucoronosyl/UDP-glucosyl transferase family protein** |
| **FLA12 (fasciclin-like arabinogalactan-protein 12)** |
| **proline-rich family protein** |
| **zinc finger (C3HC4-type RING finger) family protein** |
| **FLA12 (fasciclin-like arabinogalactan-protein 12)** |
| **FLA12 (fasciclin-like arabinogalactan-protein 12)** |
| **AHA1 (ARABIDOPSIS H+ ATPASE 1); ATPase** |
| **bZIP family transcription factor** |
| **zinc finger (C3HC4-type RING finger) family protein** |
| **VIM5 (VARIANT IN METHYLATION 5); protein binding / zinc ion binding** |
| **XCP2 (XYLEM CYSTEINE PEPTIDASE 2); cysteine-type peptidase/ peptidase** |
| **dehydration-responsive protein-related** |
| **FRA8 (FRAGILE FIBER8); transferase** |
| **ATNAP4 (ARABIDOPSIS THALIANA NON-INTRINSIC ABC PROTEIN 4)** |
| **F-box family protein-related** |
| **GUT2; catalytic** |
| **FLA12 (fasciclin-like arabinogalactan-protein 12)** |
| **ABIL1 (ABI-1-LIKE 1)** |
| **MAT2/SAM-2 (S-adenosylmethionine synthetase 2); methionine adenosyltransferase** |
| **O-acetyltransferase family protein** |
| **FAH1 (FERULATE-5-HYDROXYLASE 1); ferulate 5-hydroxylase** |
| **structural constituent of ribosome** |
| **4CL1 (4-COUMARATE:COA LIGASE 1); 4-coumarate-CoA ligase** |
| **ATNEK5; kinase** |
| **metal ion binding** |
| **aspartyl protease family protein** |
| **peroxidase, putative** |
| **GATL1/GLZ1/PARVUS (GALACTURONOSYLTRANSFERASE-LIKE 1); polygalacturonate 4-alpha-galacturonosyltransferase/ transferase, transferring glycosyl groups / transferase, transferring hexosyl groups** |
| **SUB1 (short under blue light 1)** |
| **CYP76C6 (cytochrome P450, family 76, subfamily C, polypeptide 6); oxygen binding** |
| **WPP2 (WPP domain protein 2)** |
| **ATNEK2; kinase** |
| **MYB85 (myb domain protein 85); DNA binding / transcription factor** |
| **protein kinase family protein** |
| **peptidoglycan-binding LysM domain-containing protein** |
| **exostosin family protein** |
| **auxin-responsive protein -related** |
| **kinesin light chain-related** |
| **chloroplast nucleoid DNA-binding protein, putative** |
| **Zinnia LXL library : Pesquet et. al, 2005** |
| **1 Amino-Cyclo-propane-1-Carboxylate Synthase [ACCS]** |
| **1.4 Benzoquinone reductase-like; Trp repressor binding protein-1** |
| **1.4 Benzoquinone reductase-like; Trp repressor binding protein-2** |
| **ABC transporter** |
| **Abscisic acid induced protein HVA-22-1** |
| **Abscisic acid induced protein HVA-22-2** |
| **ADP/ATP carrier protein** |
| **ADP/ATP translocator** |
| **Alpha-L-ArabinoFuranosidase** |
| **Annexin-1,2** |
| **Argonaute** |
| **C-4 Sterol Methyl Oxidase [SMO]** |
| **Caffeoyl-CoA O-methyltransferase-1 [CCoAOMT-1]** |
| **Caffeoyl-CoA O-methyltransferase-2 [CCoAOMT-2]** |
| **Caffeoyl-CoA O-methyltransferase-3 [CCoAOMT-3]** |
| **CALMODULIN-1,2,3** |
| **CALRETICULIN-1** |
| **CALRETICULIN-2** |
| **CDK5 Activator Binding Protein** |
| **Cks1 protein / putative cyclin-dependent kinase regulatory subunit** |
| **Cucumisin** |
| **Cyclin Dependent Kinase protein kinase [CDC2d]** |
| **Cysteine Proteinase-01 [CP-1]** |
| **Cysteine Proteinase-02 [CP-2]** |
| **Cysteine Proteinase-03 [CP-3]** |
| **Cysteine Proteinase-04 [CP-4]** |
| **Cysteine Proteinase-05 [CP-5]** |
| **Cysteine Proteinase-06 [CP-6]** |
| **Cysteine Proteinase-07 [CP-7]** |
| **Cysteine Proteinase-08 [CP-8]** |
| **Cysteine Proteinase-09 [CP-9]** |
| **Cysteine Proteinase-10 [CP-10]** |
| **Cysteine Proteinase-11 [CP-11]** |
| **Cysteine Proteinase-12 [CP-12]** |
| **CYTOCHROME B5-1** |
| **CYTOCHROME B5-2** |
| **Dehydration Induced Protein RD22** |
| **DNA binding protein** |
| **DnaJ Chaperonine** |
| **DolichyldiphosphoOligoSaccharide protein glycotransferase [OST]** |
| **Endonuclease** |
| **Endonuclease** |
| **Expansin-1** |
| **Expansin-2** |
| **Expansin-3** |
| **Expansin-4** |
| **Expansin-5** |
| **Gibberellic Acid Stimulated Transcript ( GAST1/GASA like) [GAST3]** |
| **Gibberellic Acid Stimulated Transcript ( RSI1 like) [GAST1]** |
| **Gibberellic Acid Stimulated Transcript (GAST1-like) [GAST2]** |
| **GTP Binding Protein SAR1A** |
| **GTP cyclohydrolase I** |
| **Histone Deacetylase [HD]** |
| **HISTONE H2A-01** |
| **HISTONE H2A-02** |
| **HISTONE H2A-03** |
| **HISTONE H2A-04** |
| **HISTONE H2B-01** |
| **HISTONE H2B-02** |
| **HISTONE H2B-03** |
| **HISTONE H2B-04** |
| **HISTONE H2B-05** |
| **HISTONE H2B-06** |
| **HISTONE H2B-07** |
| **HISTONE H2B-08** |
| **HISTONE H2B-09** |
| **HISTONE H2B-10** |
| **HISTONE H2B-11** |
| **HISTONE H3-01** |
| **HISTONE H3-02** |
| **HISTONE H3-03** |
| **HISTONE H4-01** |
| **HISTONE H4-02** |
| **HISTONE H4-03** |
| **HISTONE H4-04** |
| **HISTONE H4-05** |
| **hydroxyproline rich glycoprotein / 60S RIBOSOMAL PROTEIN L14** |
| **Kinetochore SKP1-like protein** |
| **Late Embryogenesis protein lea5 like** |
| **MAGO NASHI protein** |
| **MAGO NASHI protein** |
| **MtN21 Nodulin like - 1** |
| **MtN21 Nodulin like - 2** |
| **MtN21 Nodulin like - 3** |
| **NADPH-ferrihemoprotein reductase** |
| **Non-symbiotic hemoglobin** |
| **Nucleolar protein** |
| **Nucleoside Transporter** |
| **Oligopeptide Transporter** |
| **Pectate Lyase** |
| **Pectin Methyl Esterase [PME]** |
| **Peroxidase** |
| **PGPD14 like RING zinc finger protein-1** |
| **PGPD14 like RING zinc finger protein-2** |
| **Phospho-transfer signaling response regulator homolog** |
| **Photoassimilate Responsive Protein PAR-1b (mRNA inducible by sucrose and salicylic acid)** |
| **Polygalacturonase** |
| **Pre-Pro-PhytoSulfokine-1** |
| **Pre-Pro-PhytoSulfokine-2** |
| **Protein Kinase CK2-like** |
| **Protein Phosphatase 2C [PP2C]** |
| **Proteinase Inhibitor / Gamma-Thionin-1 [PIGT-1]** |
| **Proteinase Inhibitor / Gamma-Thionin-3 [PIGT-3]** |
| **Proteinase Inhibitor / Gamma-Thionin-4 [PIGT-4]** |
| **Proteinase Inhibitor / Gamma-Thionin-5 [PIGT-5]** |
| **Ribonuclease I** |
| **RNA polymerase II 10th largest Subunit-1 [RP10-1]** |
| **RNA polymerase II 10th largest Subunit-2 [RP10-2]** |
| **RNA polymerase II 5th largest Subunit [RP5]** |
| **S-adenosyl-L-methionine:salicylate carboxyl methyltransferase** |
| **Signal sequence processing protein** |
| **Single stranded DNA binding protein / Replication protein A2** |
| **Small Nuclear Ribonucleoprotein D2 [SNuRP]** |
| **Small Nuclear Ribonucleoprotein E [SNuRP]** |
| **Small Nuclear Ribonucleoprotein U1a [SNuRP]** |
| **Stress Induced Protein sti1** |
| **TED4-1** |
| **TED4-2** |
| **TED4-3** |
| **TED4-4** |
| **TED4-5** |
| **Thymidylate Kinase** |
| **Trans-Cinnamate-4-monooxygenase/hydroxylase [C4H]** |
| **Transposase-1** |
| **Transposase-2** |
| **Transposase-3** |
| **Ubiquitin / Ribosomal protein CEP52-1** |
| **Ubiquitin Conjugating Enzyme E2-1** |
| **Ubiquitin Conjugating Enzyme E2-2** |
| **Ubiquitin Conjugating Enzyme E2-3** |
| **Unknown Gene 01 [UG-1]** |
| **Unknown Gene 02 [UG-2]** |
| **Unknown Gene 03 [UG-3]** |
| **Unknown Gene 04 [UG-4]** |
| **Unknown Gene 05 [UG-5]** |
| **Unknown Gene 06 [UG-6]** |
| **Unknown Gene 07 [UG-7]** |
| **Unknown Gene 08 [UG-8]** |
| **Unknown Gene 09 [UG-9]** |
| **Unknown Gene 10 [UG-10]** |
| **Unknown Gene 11 [UG-11]** |
| **Unknown Gene 12 [UG-12]** |
| **Unknown Gene 13 [UG-13]** |
| **Unknown Gene 14 [UG-14]** |
| **Unknown Gene 15 [UG-15]** |
| **Unknown Gene 16 [UG-16]** |
| **Unknown Gene 17 [UG-17]** |
| **Unknown Gene 18 [UG-18]** |
| **Unknown Gene 19 [UG-19]** |
| **Unknown Gene 20 [UG-20]** |
| **Unknown Gene 22 [UG-22]** |
| **Unknown Gene 23 [UG-23]** |
| **Unknown Gene 24 [UG-24]** |
| **Unknown Gene 25 [UG-25]** |
| **Unknown Gene 26 [UG-26]** |
| **Unknown Gene 28 [UG-28]** |
| **Unknown Gene 30 [UG-30]** |
| **Unknown Gene 31 [UG-31]** |
| **Unknown Gene 32 [UG-32]** |
| **Unknown Gene 33 [UG-33]** |
| **Unknown Gene 34 [UG-34]** |
| **Unknown Gene 35 [UG-35]** |
| **Unknown Gene 36 [UG-36]** |
| **Unknown Gene 37 [UG-37]** |
| **Unknown Gene 38 [UG-38]** |
| **Unknown Gene 39 [UG-39]** |
| **Unknown Gene 40 [UG-40]** |
| **Unknown Gene 41 [UG-41]** |
| **Unknown Gene 42 [UG-42]** |
| **Unknown Gene 43 [UG-43]** |
| **Unknown Gene 44 [UG-44]** |
| **Unknown Gene 46 [UG-46]** |
| **Unknown Gene 47 [UG-47]** |
| **Unknown Gene 48 [UG-48]** |
| **Unknown Gene 49 [UG-49]** |
| **Unknown Gene 50 [UG-50]** |
| **Unknown Gene 51 [UG-51]** |
| **Unknown Gene 52 [UG-52]** |
| **Unknown Gene 53 [UG-53]** |
| **Unknown Gene 55 [UG-55]** |
| **Unknown Gene 56 [UG-56]** |
| **Unknown Gene 57 [UG-57]** |
| **Unknown Gene 58 [UG-58]** |
| **Unknown Gene 59 [UG-59]** |
| **Unknown Gene 60 [UG-60]** |
| **Unknown Gene 61 [UG-61]** |
| **Unknown Gene 62 [UG-62]** |
| **Unknown Gene 63 [UG-63]** |
| **Unknown Gene 64 [UG-64]** |
| **Unknown Gene 65 [UG-65]** |
| **Unknown Gene 66 [UG-66]** |
| **Unknown Gene 67 [UG-66]** |
| **Unknown Gene 68 [UG-68]** |
| **Unknown Gene 69 [UG-69]** |
| **Unknown Gene 70 [UG-70]** |
| **Unknown Gene 71 [UG-71]** |
| **Xylanase / Proline-rich glycoprotein** |
| **40S RIBOSOMAL PROTEIN S15** |
| **40S RIBOSOMAL PROTEIN S23-01** |
| **40S RIBOSOMAL PROTEIN S23-02** |
| **40S RIBOSOMAL PROTEIN S23-04** |
| **40S RIBOSOMAL PROTEIN S23-05** |
| **40S RIBOSOMAL PROTEIN S23-06** |
| **40S RIBOSOMAL PROTEIN S23-07** |
| **40S RIBOSOMAL PROTEIN S23-08** |
| **40S RIBOSOMAL PROTEIN S23-09** |
| **40S RIBOSOMAL PROTEIN S23-10** |
| **40S RIBOSOMAL PROTEIN S23-11** |
| **40S RIBOSOMAL PROTEIN S23-12** |
| **40S RIBOSOMAL PROTEIN S25-01** |
| **40S RIBOSOMAL PROTEIN S25-02** |
| **40S RIBOSOMAL PROTEIN S25-03** |
| **40S RIBOSOMAL PROTEIN S3a** |
| **40S RIBOSOMAL PROTEIN S6-1** |
| **60S RIBOSOMAL PROTEIN L29** |
| **60S RIBOSOMAL PROTEIN L12** |
| **60S RIBOSOMAL PROTEIN L17** |
| **60S RIBOSOMAL PROTEIN L17-1** |
| **60S RIBOSOMAL PROTEIN L17-2** |
| **60S RIBOSOMAL PROTEIN L18** |
| **60S RIBOSOMAL PROTEIN L27A** |
| **60S RIBOSOMAL PROTEIN L28** |
| **60S RIBOSOMAL PROTEIN L31** |
| **60S RIBOSOMAL PROTEIN L32** |
| **60S RIBOSOMAL PROTEIN L34-1** |
| **60S RIBOSOMAL PROTEIN L34-2** |
| **60S RIBOSOMAL PROTEIN L35a** |
